# Formation damage simulation of a multi-fractured horizontal well in a tight gas/shale oil formation

**DOI:** 10.1007/s13202-022-01544-8

**Published:** 2022-07-19

**Authors:** Dung Bui, Tan Nguyen, Thanh Nguyen, Hyunsang Yoo

**Affiliations:** 1 New Mexico Tech, Socorro, New Mexico USA; 2 Computer Modelling Group, Calgary, Canada

**Keywords:** Formation damage, Fracture fluid leak-off, Shut-in well damage, Hydraulic fracture simulation, Multi-fractured horizontal well, Tight formations

## Abstract

Formation damage in drilling comes from drilling fluid invasion due to high differential pressure between a wellbore and the formation. This mechanism happens with fracture fluid invasion of multi-fractured horizontal wells in tight formations. Some multi-fractured wells show production rates and cumulative productions far lower than expected. Those damaged wells may sustain further impact such as well shutting due to unexpected events such as the COVID-19 outbreak and then experience a further reduction in cumulative production. This paper focuses on the root causes of formation damage of fractured wells and provides possible solutions to improve production. A simulation study was conducted using Computer Modelling Group software to simulate formation damage due to fracture fluid invasion and well shut-in. Simulation results revealed that the decrease in cumulative hydrocarbon production due to leak-off and shut-in of the simulated well could range from 20 to 41%, depending on different conditions. The results showed that the main causes are high critical water saturation of tight formations, low drawdown, and low residual proppant permeability under formation closure stress. The sensitivity analysis suggests two feasible solutions to mitigate formation damage: optimizing drawdown during production and optimized proppant pack permeability of the hydraulic fracturing process. Optimizing pressure drawdown is effective in fixing leak-off damage, but it does not mitigate shut-in damage. Formation damage due to shut-in should be prevented in advance by using an appropriate proppant permeability. These key findings enhance productivity and improve the economics of tight gas and shale oil formations.

## Introduction and Literature Review

According to the Energy Information Administration (EIA) in its Natural Gas Monthly report, February 2021, the US natural gas production in 2020 was about 34.4 trillion cubic feet. It was the second-highest annual gas production recorded and roughly 10% higher than the US total natural gas consumption in that year. (The highest production recorded was 35 trillion cubic feet in 2019.) The EIA also reported that about 2.67 billion barrels of crude oil were produced directly from shale oil formations in the USA. This production was about 65% of total US crude oil produced in 2020. Tight gas and shale oil formations contribute a vital role in providing sufficient hydrocarbons for energy consumption and ensuring energy security in the USA.

Due to the low permeability of tight formations, most of the hydrocarbon production in the USA from 2010 to 2019 was based on the success of two crucial techniques: horizontal drilling and hydraulic fracturing. With recent technological improvements, a horizontal well can be drilled up to five miles in lateral length and with 20 to more than 200 fracture stages. Generally, the hydraulic fracturing process consists of four steps: (1) pumping a pad fluid without proppant to initiate fracture, (2) main treatment schedule: pumping fluid with proppant to increase proppant concentration step by step, (3) displacement of the proppant-laden slurry to the desired fracture length, and (4) stop pumping to allow fracture fluid leak-off into the formation and formation closure on proppant, according to Miskimins et al. (2019).

Due to high injection pressure of the hydraulic fracturing process, fracturing fluid invades the porous medium. That phenomenon causes formation damage, which reduces the effective permeability of the stimulated zone (Ding et al. 2013, Qutob et al. 2015, Liang et al. 2017). In addition, the oil and gas industry has undergone an unprecedented pandemic. Many production wells in tight gas/shale oil formations have been shut in a long time. After shut-in, some of these wells experience a reduction in production with several possible reasons (Garduno and King 2020).

There has not been much research about quantifying the production decrease due to formation damage associated with hydraulic fracturing and shut-in damage in tight gas/shale oil formations. Therefore, this paper concentrates on (1) simulation of hydraulic fracture propagation in an example tight reservoir, (2) quantifying production decrease due to formation damage, (3) estimating production decrease due to shut-in, (4) proposing potential solutions to reduce formation damage, therefore improving hydrocarbon recovery.

To accomplish the above tasks, the authors used a Computer Modelling Group (CMG) reservoir simulator which features iterative coupling of reservoir fluid flow and geomechanics deformation (Tran et al. 2009a). A dual permeability IMEX model is used, which allows each grid block to have two porosity systems, one called matrix porosity and the other called fracture porosity. This model allows the inter-block flow to occur from fracture-to-fracture, matrix-to-fracture, and matrix-to-matrix (CMG tutorial 2020). After hydraulic fracturing, formation damage due to water-based fracturing fluid invasion was evaluated through water saturation increase and gas/oil (non-wetting phase) relative permeability decrease (Liang et al. 2017). As the dual permeability model is used, the water saturation term generally refers to both matrix and fracture water saturations, unless specified. A correlation between water saturation and permeability presented by Wu et al. (2009) was applied to calculate reservoir permeability in the damaged zone.

1 $$\frac{{k_{{\text{d}}} }}{k} = 1 + \left( {\alpha - 1} \right)\left( {\frac{{S_{{\text{w}}} - S_{{{\text{wi}}}} }}{{1.0 - S_{{{\text{gc}}}} - S_{{{\text{wi}}}} }}} \right)^{{b_{w} }}$$

where $k$ is the absolute permeability, $k_{{\text{d}}}$ is the damage permeability profile in the invaded zone, and $b_{{\text{w}}}$ is the exponent of relative permeability. $S_{{\text{w}}}$, $S_{{{\text{wi}}}}$, and $S_{{{\text{gc}}}}$ are water saturation, initial water saturation, and critical gas saturation, respectively. The coefficient $\alpha$ denotes the formation damage factor, which is the ratio of the return permeability measured from laboratory experiments on a core to the undamaged reservoir permeability *k*. Although Wu et al. did not mention specific values of $\alpha$ and $b_{{\text{w}}}$ coefficients since they are dependent on each core sample, the authors conducted a fitting technique between the empirical equation and the presented water saturation distribution near wellbore versus damaged permeability profile in Wu et al.’s publication to obtain values of $\alpha$ and $b_{{\text{w}}}$ coefficients of 0.033 and 1.58, respectively. Then, these values were used in numerical simulation to quantify how severe the formation was damaged by facture fluid invasion. The fitting process is shown in “Appendix 1”.

**Fig. 1:**
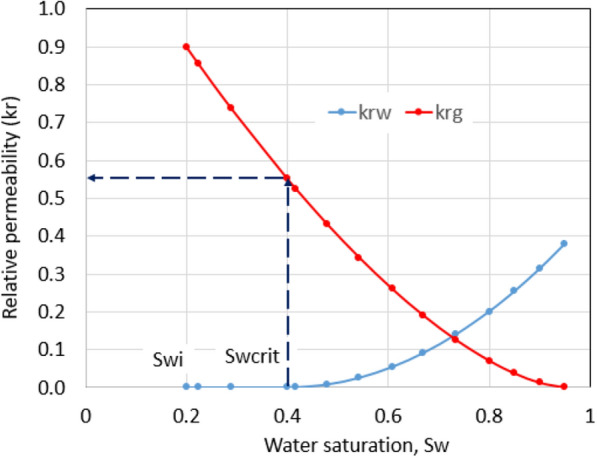
Water saturation and relative permeability

The fracture fluid invasion in some tight formations cannot be removed during flowback (Qutob et al. 2015). This phenomenon relates to the phase trapping mechanism and critical water saturation as follows:

The phase trapping mechanism is explained in Fig. 1. Many unconventional formations are water undersaturated, which have the initial water saturation (*S*_wi_) lower than the critical water saturation (*S*_wcrit_). *S*_wcrit_ is defined as the minimum water saturation at which water first becomes movable. If fracture fluid invades a water undersaturated reservoir, it will increase the reservoir water saturation from *S*_wi_ to *S*_wcrit_ and then exceed *S*_wcrit_. Fracture fluid invasion is shown by the increase in water saturation in both matrix and the fracture systems. A part of the water saturation after hydraulic fracturing which exceeds *S*_wcrit_ can be removed during flowback, but the rest becomes unmovable below *S*_wcrit_.

The *S*_wcrit_ in Fig. 1 is 0.4 and higher than the *S*_wi_ of 0.2. As fracture fluid starts to invade the formation, water saturation first increases from its initial value of 0.2 to the critical value of 0.4, and then it exceeds the critical value and could go up to the endpoint, which equals (1—critical gas saturation). This behavior causes gas relative permeability to decrease from 0.9 to 0.55 as shown in Fig. 1. Water does not move at water saturation that equals *S*_wcrit_ because the water relative permeability is 0, so it cannot be removed during flowback. This phenomenon is called phase trapping. If water saturation increases higher than *S*_wcrit_, gas relative permeability is reduced further. For example, gas relative permeability is only 0.27 at the water saturation of 0.6 as shown in Fig. 1.

In a water-wet two-phase flow system, hydrocarbon production is calculated using the effective permeability of the non-wetting phase. Effective permeability is a product of absolute permeability and relative permeability. Thus, formation damage in the invaded zone is the combination of reservoir permeability reduction calculated by Eq. (1) and relative permeability reduction due to phase trapping. The phase trapping mechanism extends the clean-up period and, therefore, reduces cumulative gas production in the future. In some formations with very high *S*_wcrit_, the formation damage due to fracture fluid invasion is much more severe, and recovery will be much less than expected.

The removal of water saturation depends on the following factors:

- The available drawdown in the production phase: High drawdown will shorten the clean-up period and thus mitigate production impairment.
- Residual proppant permeability: critical factor to accelerate the removal of blocking fracture fluid. A high proppant conductivity provides a better clean-up.
- The difference between *S*_wi_ and *S*_wcrit_: In a tight formation which has a significant difference between *S*_wi_ and *S*_wcrit_, the best practice of minimizing formation damage is to keep altered water saturation below *S*_wcrit_ as much as possible.

Knowing the *S*_wcrit_ of the developing formation is the first step of quantifying formation damage due to the phase trapping mechanism. *S*_wcrit_ is usually determined by special core measurement or using analytical models. Chen et al. (2017) presented an analytical model to predict *S*_wcrit_, and Su et al. (2020) improved the model by introducing the concept of $\eta$, dead or stationary water (DSW) coefficient—immovable water in the special pores that cannot be removed under flow conditions. These two models were able to predict *S*_wcrit_ with an absolute error from 0.3 to 2%. In Su's new model, the volume of immobile water and the total pore were calculated as follows:

2 $$\begin{aligned} V_{{{\text{wcrit}}}} & = N \mathop \int \limits_{{r_{{{\text{mins}}}} }}^{{r_{{{\text{cs}}}} }} \pi r_{{\text{s}}}^{2} L_{{\text{s}}} \left( {r_{{\text{s}}} } \right)f_{{\text{s}}} \left( {r_{{\text{s}}} } \right){\text{d}}r \\ & \quad + N\mathop \int \limits_{{r_{{{\text{cs}}}} }}^{{r_{{{\text{maxs}}}} }} \pi \left[ {r_{{\text{s}}}^{2} - \left( {r_{{\text{s}}} - \delta } \right)^{2} } \right]L_{{\text{s}}} \left( {r_{{\text{s}}} } \right)f_{{\text{s}}} \left( {r_{{\text{s}}} } \right){\text{d}}r \\ & \quad + \eta N\mathop \int \limits_{{r_{{{\text{mins}}}} }}^{{r_{{{\text{maxs}}}} }} r_{{\text{s}}}^{2} L_{{\text{s}}} \left( {r_{{\text{s}}} } \right)f_{{\text{s}}} \left( {r_{{\text{s}}} } \right){\text{d}}r \\ \end{aligned}$$

3 $$V_{{{\text{total}}}} = \left( {1 + \eta } \right)N\mathop \int \limits_{{r_{{{\text{mins}}}} }}^{{r_{{{\text{maxs}}}} }} r_{{\text{s}}}^{2} L_{{\text{s}}} \left( {r_{{\text{s}}} } \right)f_{{\text{s}}} \left( {r_{{\text{s}}} } \right){\text{d}}r$$

where $V_{{{\text{wcrit}}}}$ is the volume of the immobile water, m^3^; $V_{{{\text{total}}}}$ is the volume of the total pore space, m^3^; *N* is the number of pores in a unit cell; $L_{{\text{s}}}$ is the tortuous flow path under effective stress, m; $f_{{\text{s}}}$ is the probability density function for pore size distribution under effective stress; $r_{{{\text{cs}}}}$ is the critical pore radius under effective stress, m; $r_{{{\text{maxs}}}}$ is the maximum pore radius under effective stress, m; $r_{{{\text{mins}}}}$ is the minimum pore radius under effective stress, m; $r_{{\text{s}}}$, is the inner radius of a certain pore under effective stress, m. $r_{{\text{s}}}$ is calculated as follows:

4 $$r_{{\text{s}}} = r\left\{ {1 - 4\left[ {\frac{{3\pi (1 - v^{2} \sigma_{{{\text{ob}}}}^{^{\prime}} }}{4E}} \right]^{\beta } } \right\}$$

where *r* is the inner radius of a certain pore, m; $\sigma_{{{\text{ob}}}}^{^{\prime}}$ is the effective overburden stress calculated by Terzaghi stress law; *E* is Young’s modulus, *v* is Poisson’s ratio, dimensionless; $\beta$ is the power-law index which is affected by the pore pressure.

By the definition of critical water saturation, it is then calculated as follows:

5 $$S_{{{\text{wcrit}}}} = \frac{{V_{{{\text{wcrit}}}} }}{{V_{{{\text{total}}}} }}$$

**Fig. 2:**
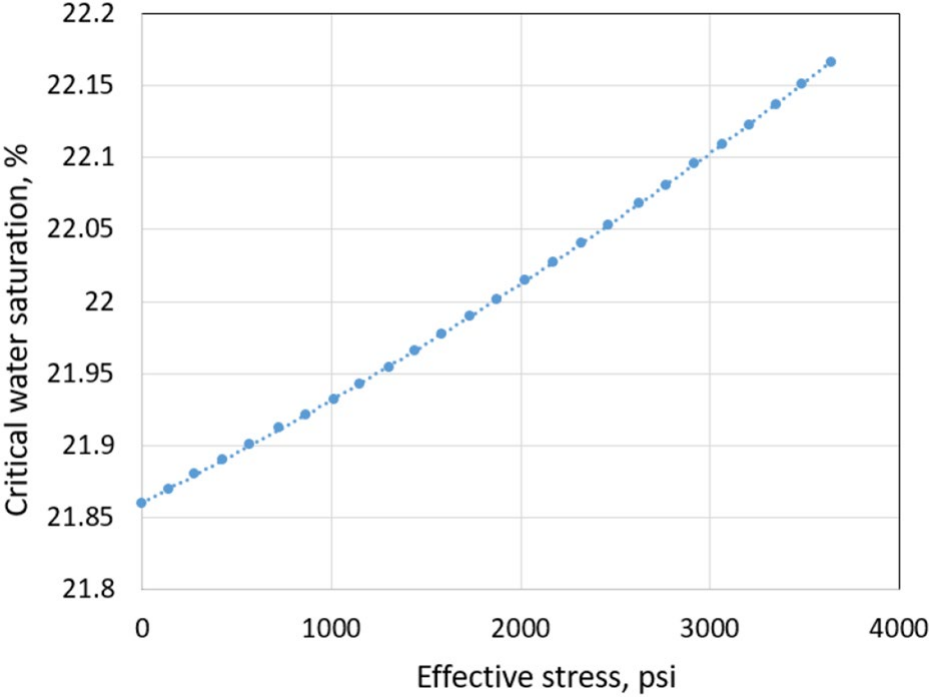
*S*_wcrit_ versus effective stress

**Fig. 3:**
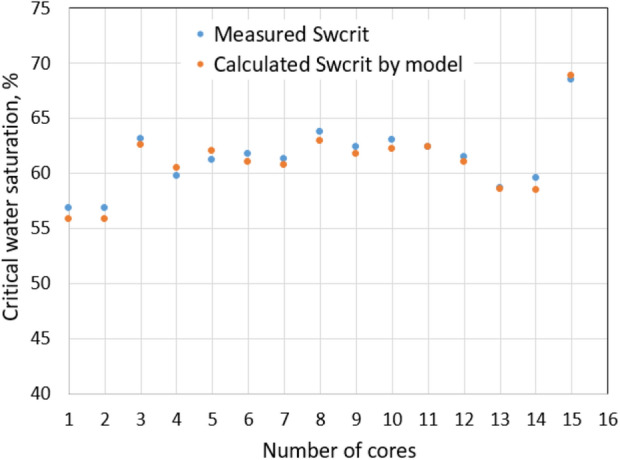
*S*_wcrit_ obtained from cores data and analytical model

Chen et al. (2017) and Su et al. (2020) models showed consistency with critical water saturation measurement of a total 30 low permeability tight sandstone cores as shown in Figs. 2 and 3.

From Eqs. (2), (3), and (5), *S*_wcrit_ is dependent on the effective stress, Young’s modulus, pore radius, DSW coefficient, and other microstructural parameters.

To quantify formation damage and production impairment caused by fracture fluid invasion, the well was produced under formation damage conditions and compared with production of the no damage condition.

Scenarios of production impairment due to well shut-in were analyzed based on two observations. First, the remaining slickwater in the fracture system imbibes into the matrix the shut-in time. It damages the absolute permeability and relative permeability of the matrix following the above phase trapping mechanisms. However, fractures are the main flowing channels in a fractured reservoir, while the matrix is the primary storage space. Because of this, shut-in damage should be a combination of both matrix permeability and fracture permeability reductions.

Many researchers have pointed out that the proppant embedment phenomenon reduces the fracture permeability due to closure stress under downhole conditions and long stress-bearing time (Wen et al. 2007; Lacy et al. 1998; Gou et al. 2017; Bandara et al. 2019; Ahamed et al. 2021). Wen et al. (2007) developed a correlation between conductivity and closure pressure and the change of conductivity with time at a certain closure pressure by regression of the experimental data. Time-dependent fracture conductivity for 20/40-mesh proppant under 60 MPa closure pressure regression is as follows:

6 $$F_{{{\text{RCD}}}} = 37.256e^{ - 0.0372*t} + C$$

where *F*_RCD_ is the long-term fracture conductivity, µm^2^*cm; t is time, day, and C is in µm^2^*cm.

Equation (6) shows that the long-term fracture conductivity changes with time under a certain closure pressure. Fracture permeability will become the same as the formation permeability when the fracture ceases to be effective. So the constant C, which is equal to the product of fracture width and formation permeability, is added in the regression. Based on Eq. (6), an initial conductivity of 45 µm^2^.cm (1476 mD.ft) will reduce to 0.43 µm^2^.cm (14 mD.ft) after 120 days. This shows a 100-fold reduction in fracture conductivity.

Interestingly, cyclic loading conditions associated with well shut-in also increase embedment, and this phenomenon has been mentioned by Lacy et al. (1998). Bandara et al. (2019) showed that the embedment could occur in any formation, whatever the type of rock, leading to fracture conductivity in a shale formation reduced by 78.05% when the closure pressure increased from 7 to 70 MPa (1015–10,150 psi) and significant permeability reduction is caused during the initial 20% of the proppant embedment process. Combining both matrix and fracture permeability reductions makes it feasible to quantify formation damage due to well shut-in using the production comparison method.

Most precedent research presented formation damage due to drilling fluid or presumed a fixed fracture geometry in 2D before quantifying the effect of fracture fluid on formation damage (He and Liu 2021; Ding et al. 2013). Others pointed out formation damage mechanisms using experimental results (Qutob and Byrne 2015; Liang et al. 2017; Cheng et al. 2022; Li et al. 2021). There is a minimal proposed solution for formation damage removal (Chen et al. 2021). Therefore, this paper provides a novel approach in the simulation of formation damage due to fracture fluid where fractures are created in 3D by applying the typical fracture treatment schedule in fields. Furthermore, the authors conducted a local sensitivity analysis technique (i.e., change one variable at a time) to propose optimal pressure drawdown and proppant permeability to mitigate formation damage. In addition, the shut-in damage phenomenon in many fractured wells in tight gas and shale oil formations was simulated in 3D, and recommendations were proposed to avoid this type of damage in advance.

## Fundamentals of the Computational Simulation Model

Basic equations for coupled fluid flow and deformation consist of two sets: one for fluid flow in porous media and another set for rock deformation. The fluid flow equations include conservation of mass and energy; the basic equations for rock deformation include deformation, strain, and stress. In the iterative coupling approach, the two sets of equations are solved separately. At a certain time step, pressure in the reservoir flow simulator is computed first and then sent to the geomechanics simulator, where displacement, strain, and stress are computed (Tran et al. 2008). The solution from the geomechanics module is then passed back to the reservoir simulation via two coupling variables—porosity and permeability. Since mass conservation depends mostly on pore volume and less on permeability, porosity is considered the primary and permeability is the secondary coupling variable. These two coupling variables are used to recompute a new pressure distribution in the reservoir simulator. Again, this new pressure is resent to the geomechanics module to recalculate deformation, strain, and stress. The above process is continued until an acceptable error tolerance is achieved (Tran et al. 2009a). The two sets of reservoir flow and geomechanics modules are as follows:

### Reservoir Flow

Mass conservation equation:

7 $$\frac{{\partial \left( {\phi^{*} \rho_{{\text{f}}} } \right)}}{\partial t} = - \nabla \cdot \left( {\rho_{{\text{f}}} v} \right) + Q_{{\text{f}}}$$

Velocity in the form of Darcy’s law:

8 $${\mathbf{v}} = - \frac{1}{\mu }{\mathbf{k}}\left( {\nabla p - \rho_{{\text{f}}} {\mathbf{g}}} \right)$$

On substituting Eq. (8) into (7), the mass flow equation in a porous medium is obtained as follows:

9 $$\frac{\partial }{\partial t}\left( {\phi^{*} \rho_{{\text{f}}} } \right) - \nabla .\left( {\rho_{{\text{f}}} \frac{{\mathbf{k}}}{\mu }.\left[ {\nabla p - \rho_{{\text{f}}} {\mathbf{g}}} \right]} \right) = Q_{{\text{f}}}$$

where $\rho_{{\text{f}}}$ is the fluid density, kg/m^3^; **u** is the velocity vector, m/s; $Q_{{\text{f}}}$ is the mass flow rate of fluid per unit volume; **k** is the absolute permeability tensor, m^2^; p is the fluid pressure, Pa; **g** is the gravitational acceleration, m/s^2^; *µ* is the fluid viscosity, Pa.s.

The porosity used in Eq. (9) of reservoir flow is called reservoir porosity which is defined as

10 $$\phi^{*} = {\text{Reservoir}}\;{\text{porosity}} = \frac{{{\text{Current}}\;{\text{pore}}\;{\text{volume}}}}{{{\text{Initial }}\;{\text{bulk}}\;{\text{volume}}}} = \frac{{V_{{\text{p}}} }}{{V_{{\text{b}}}^{0} }}$$

On the contrary, the true porosity calculated by the geomechanics module is defined as

11 $$\phi = {\text{True}}\;{\text{porosity}} = \frac{{{\text{Current}}\;{\text{pore}}\;{\text{volume}}}}{{{\text{Current}}\;{\text{bulk}}\;{\text{volume}}}} = \frac{{V_{{\text{p}}} }}{{V_{{\text{b}}} }}$$

The relationship between these two porosities is

12 $$\phi^{*} = \left( {1 - \varepsilon_{{\text{V}}} } \right)\phi$$

where $\varepsilon_{{\text{V}}} = \left( {V_{{\text{b}}}^{0} - V_{{\text{b}}} } \right)/V_{{\text{b}}}^{0}$ is the volumetric strain.

Reservoir porosity is not only a function of pressure and temperature, but also a function of mean total stress which is related to the deformation caused by changes in pressure and temperature according to Tran et al. (2002). Thus, it is used to couple reservoir flow with the geomechanics module in a two-way coupling computation.

### Geomechanics Module

The basic equations to calculate geomechanics parameters include force equilibrium, the strain–displacement relation, and the constitutive law for the solid rock.

For a stressed body to remain at rest, the total force acting on the body must be 0. Force equilibrium equation:

13 $$\nabla \cdot { }{{\varvec{\upsigma}}} - { }\rho_{r} {\mathbf{B}} = 0$$

where $\rho_{r}$ is the solid grain density, kg/m^3^; **B** is the body force acting throughout a unit volume; $\nabla \cdot { }{{\varvec{\upsigma}}}$ is the surface force including normal and shear force acting on surfaces of a unit volume.

Infinitesimal strain and displacement relation:

14 $${{\varvec{\upvarepsilon}}} = \frac{1}{2}\left[ {\nabla {\mathbf{u}} + \left( {\nabla {\mathbf{u}}} \right)^{{\text{T}}} } \right]$$

where ${{\varvec{\upvarepsilon}}}$ is the strain tensor and **u** is the displacement vector.

The effective stress is expressed as a function of strain by the constitutive laws, as follows:

Constitutive relation for solid rock:

15 $${\mathbf{\sigma^{\prime}}} = \left\{ {\mathbf{C}} \right\}\left\{ {{\varvec{\upvarepsilon}}} \right\}$$

where **C** is the tangential stiffness tensor.

To estimate fluid pressure required to initiate hydraulic fracture, the effective stress law of Terzaghi, 1925, is introduced as follows:

16 $${{\varvec{\upsigma}}} = {\mathbf{\sigma^{\prime}}} + {{\varvec{\upalpha}}}{ }p{ }{\mathbf{I}}$$

where ${{\varvec{\upalpha}}}$ is Biot’s constant which has a value in the range of 0.7–1.0, **I** is the unit tensor, *p* is the fluid pressure.

By substituting Eqs. (14), (15), and (16) into Eq. (13), the displacement equation in an isothermal case can be obtained:

17 $$\nabla \cdot \left[ {{\mathbf{C}}:\frac{1}{2}\left( {\nabla {\mathbf{u}} + \left( {\nabla {\mathbf{u}}} \right)^{{\text{T}}} } \right)} \right] = \rho_{{\text{r}}} {\mathbf{B}} - { }\nabla \cdot \left( {\alpha p} \right) {\mathbf{I}}$$

Equation (9) is solved for pressure p in a certain time step in the iterative approach. The value of p then is used in Eq. (17) to solve for the displacement **u**. After the displacement **u** is solved, strain tensor ${{\varvec{\upvarepsilon}}}$ and stress tensor ${{\varvec{\upsigma}}}$ can be computed using Eqs. (14) and (15). Based on the updated geomechanics information, new values of p and then displacement are recomputed. This process is repeated until convergence is achieved.

### Barton–Bandis Model of Fracture Permeability

**Fig. 4:**
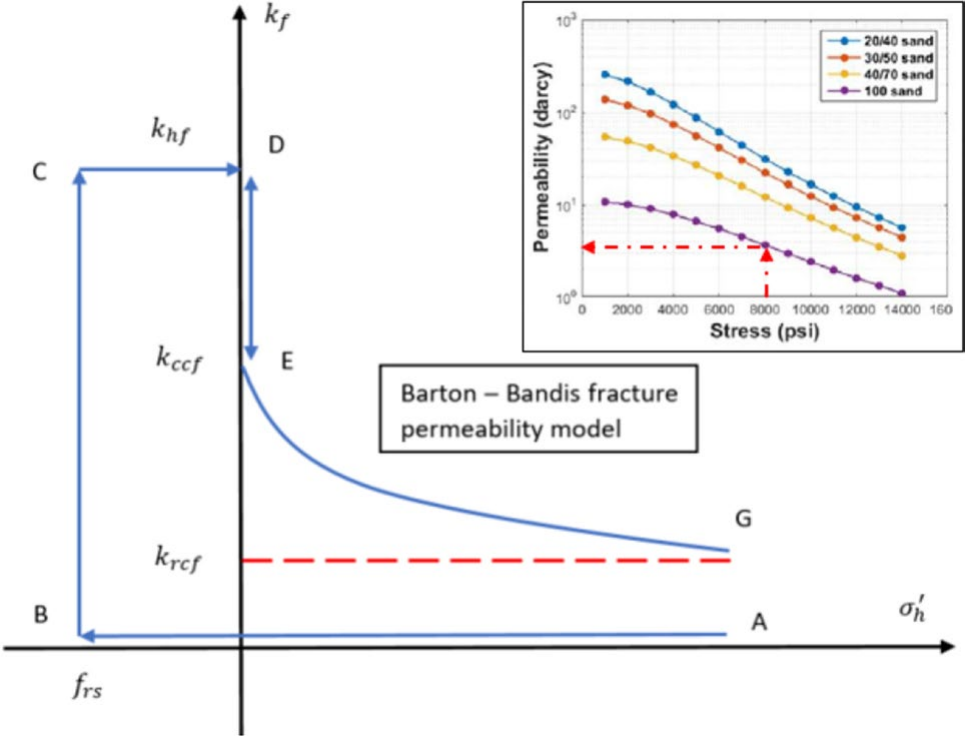
Modified of Barton–Bandis fracture permeability model

A fracture permeability based on the modification of the Barton–Bandis model (1985) is used in CMG geomechanics simulator as shown in Fig. 4 (Tran et al. 2009b). In the beginning, effective minimum horizontal stress $\left( {\sigma_{{\text{h}}}^{^{\prime}} } \right)$ is at point A. Fractures initiate when $\sigma_{{\text{n}}}^{^{\prime}}$ reduces from its initial value to tensile strength ($f_{{{\text{rs}}}} )$ at point B, and the stimulated zone has permeability that equals proppant permeability ($k_{{{\text{hf}}}} )$. Once pumps shut, and fractures start to close under closure stress. $\sigma_{{\text{h}}}^{^{\prime}}$ increases from point C to point D, and permeability of the fractured zone decreases from $k_{{{\text{hf}}}}$ at point D to closure permeability ($k_{{{\text{ccf}}}} )$ at point E. With the mechanical support of proppant, fractures do not close entirely, but they will lose their conductivities and be forced to close gradually (Huang et al. 2019). As fractures close and proppant pore structures compact, the effective permeability of the stimulated zone is reduced to a lower value called residual fracture permeability ($k_{{{\text{rcf}}}} )$ at point G. According to Huang et al. (2019), the permeability of 100-mesh sand under 8000 psi stress is $10^{0.25}$ Darcy or 1700 mD instead of 10,000 mD under zero stress as shown in Fig. 4.

### Computational Simulation Setup

CMG’s IMEX reservoir simulation software was used to simulate the induced hydraulic fractures and flows of a natural fractured tight formation. The reservoir depth is at 8000 ft (2438 m) with 5400 ft (1646 m) on the x-axis, 2010 ft (612 m) on the y-axis, and 100 ft (30 m) on the z-axis (reservoir thickness is 100 ft). This formation is discretized by 94 × 201 × 10 grid blocks. The reservoir and rock properties used in the simulation are given in Table 1.

**Table 1:** Reservoir and rock properties

Top formation true vertical depth, TVD	8000 ft (2438 m)
Reservoir permeability, *k*	0.01 mD
Reservoir porosity, $\phi^{*}$	0.05
Initial pore pressure, *P*	2800 psi
Initial water saturation, $S_{{{\text{wi}}}}$	0.2
Critical gas saturation, $S_{{{\text{gc}}}}$	0.05
Young’s modulus, *E*	7E06 psi
Poisson’s ratio, $v$	0.25
Total vertical stress, $\sigma_{{\text{V}}}$	8000 psi
Total minimum horizontal stress, $\sigma_{{\text{h}}}$	4533 psi

The chosen rock properties such as porosity and permeability are typical for tight rock formations. They can also be considered as the high-end values of shale formations. Thus, to compare the simulation results of tight gas and shale oil, all rock properties were kept the same for both types of formations. Note that total vertical stress of 8000 psi was calculated based on assuming an overburden stress gradient of 1 psi/ft. Effective vertical stress computed by Terzaghi effective stress law is 5200 psi. Then, the effective minimum horizontal stress was computed by using Hubert and Willis equation (Eq. 18):

18 $$\sigma_{{\text{h}}}^{^{\prime}} = \frac{v}{1 - v}*\sigma_{{\text{v}}}^{^{\prime}}$$

where $\sigma_{{\text{h}}}^{^{\prime}}$ and $\sigma_{{\text{v}}}^{^{\prime}}$ are effective minimum horizontal and effective vertical stress, respectively, and $v$ is Poisson’s ratio. As effective minimum horizontal stress is 1733 psi, Terzaghi effective stress law was applied again to compute the total minimum horizontal stress of 4533 psi.

**Fig. 5:**
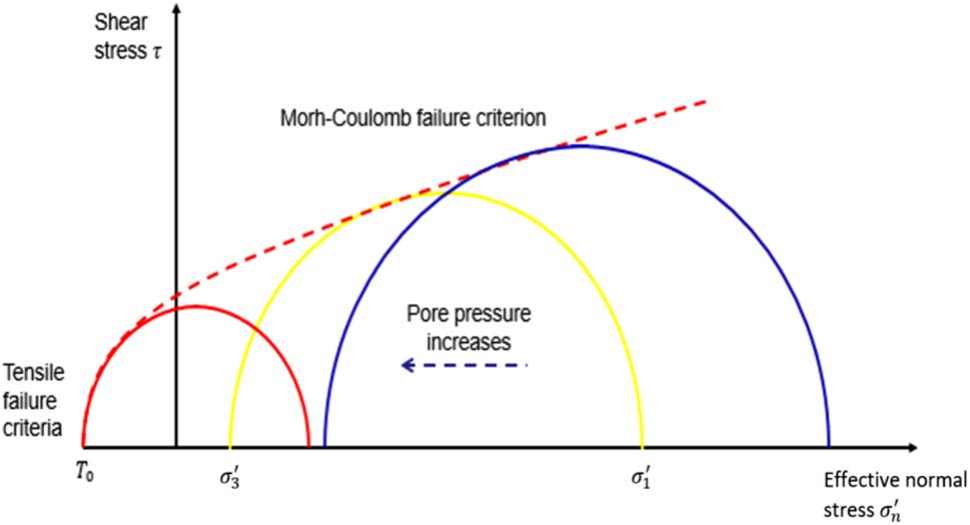
Tensile fracture using Mohr–Coulomb approach

A 5000-ft-long (1524 m) horizontal well was drilled at the center of the reservoir. Then, it was hydraulically fractured by injecting 70 bpm (16,026 m^3^/D) water-based fluid in 2 h for each stage. There are ten fracture stages with fracture spacing of 500 ft. To create the hydraulic fracture, effective stress law by Terzaghi (1925) and Mohr–Coulomb stress model (Labuz and Zang 2012) were combined in CMG simulation. In the beginning, the in situ stress state was not under the failure condition. When fracture fluid was injected into the formation, it increased pore pressure and, therefore, decreased effective stress. For tensile fracture mode, which is the most common failure type in hydraulic fracturing, the stress condition is given as in Eq. (19):

19 $$\sigma_{{\text{h}}}^{^{\prime}} = \sigma_{{\text{h}}} - P > T_{0}$$

where $\sigma_{{\text{h}}} \;{\text{and}}\;\sigma_{{\text{h}}}^{^{\prime}}$ are the total and effective minimum horizontal stress, respectively. P is the pore pressure, and $T_{0}$ is the tensile rock strength. As shown in Fig. 5, pore pressure increase shifts the Mohr circle in the Mohr–Coulomb stress model to the left-hand side (Cuss et al. 2015). If the pore pressure increases to a certain value that effective minimum horizontal stress drops below the rock tensile strength, the fracture criterion is satisfied and hydraulic fracture is initiated and propagated perpendicular to the minimum horizontal stress direction.

This study intends to compare production between non-damaged and damaged formations caused by fluid leak-off and well shut-in. It is also important to determine in which conditions the formation damage becomes severe and unacceptable. Because of that, the simulation was conducted in two different conditions as shown in Table 2: normal and extreme conditions. *S*_wcrit_, residual proppant permeability, and pressure drawdown were chosen to be investigated based on phase trapping mechanism and slickwater invasion removal as presented in the literature review section.

**Table 2:** Input data of normal and extreme conditions

	Normal conditions	Extreme conditions
Critical water saturation	0.4	0.6
Residual proppant permeability	1000 mD	100 mD
Pressure drawdown during production	600 psi	200 psi

*S*_wcrit_ of the normal conditions is 0.4 based on Su et al. (2020) proposed model. Figure 3 also shows that *S*_wcrit_ value can be up to 0.6–0.7. Thus, *S*_wcrit_ of 0.6 is used in the simulation of the extreme conditions.

In the normal conditions, it is considered that 4000 mD proppant permeability is pumped into the reservoir along with slickwater, and residual permeability under closure stress is 1000 mD based on the modified Barton–Bandis fracture permeability model as stated above. Under high closure stress, the proppant can be crushed and lose its conductivity quickly. Thus, the simulation of extreme conditions considers the residual proppant pack permeability of 100 mD.

Remedy of formation damage depends on available drawdown during production as discussed in the literature review. It also suggests that a low drawdown pressure does not help in removing blocking water but extends clean-up time and further reduces cumulative production.

Therefore, 600 psi and 200 psi drawdown are used in the normal and extreme conditions, respectively, to investigate the effect of drawdown on production.

## Results and Discussion

### Leak-Off Damage in Tight Gas Formation

#### Gas Production Impairment Under Normal Conditions

**Fig. 6:**
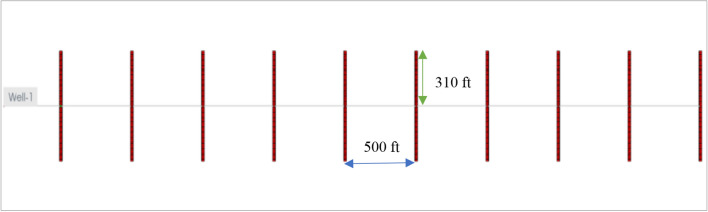
Geometry of 10 fracture stages in the tight gas well

**Fig. 7:**
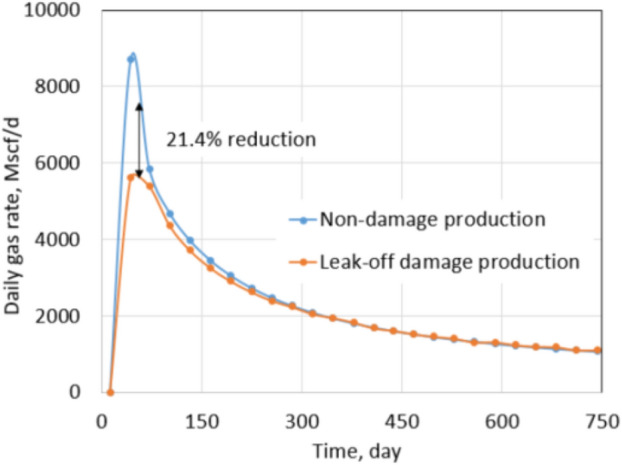
Daily gas rate comparison between non-damaged and leak-off damaged production under normal conditions

**Fig. 8:**
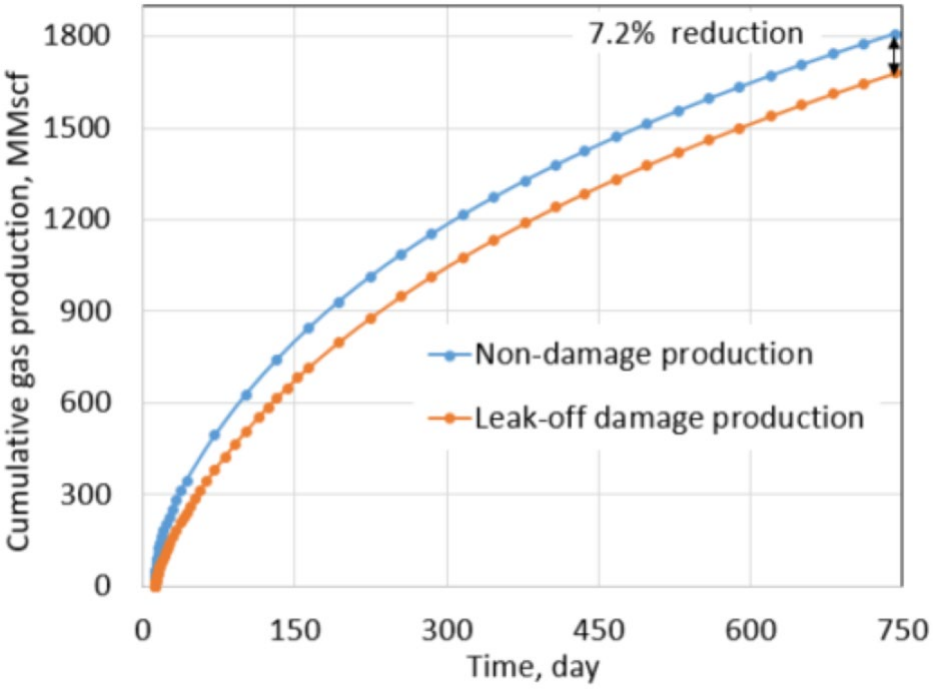
Cumulative production comparison between non-damaged and leak-off damaged under normal conditions

At first, a horizontal well was drilled at the center of the reservoir, and then it was completed with ten hydraulic fractures. Each fracture stage was created by injecting 70 bpm (16,026 m^3^/D) of fracture fluid in 2 h. Figure 6 shows the fracture half-lengths and fracture spacing of the ten stages from the aerial view of the x–y plane. Since the injection rate and pumping time are the same for the ten stages, the fracture half-lengths are the same and equal to 310 ft (95 m) for each stage. Due to the fracture closure mechanism, the proppant pack permeability (fracture permeability) changes from 4000 to 1000 mD. For slickwater hydraulic fracturing, it is common to assume that the pumping time for each stage is 2 h, and the total shut-in time is 10 days for each stage. After 10 days of a shut-in, the well is forced to flowback and produce under 600 psi drawdown in 2 years. Ideally, a non-damaged formation should have: (1) the permeability of the fractured zone equal to the proppant permeability, and (2) no water saturation increase in the formation. In other words, all fracturing fluid is used to break the formation, and water saturation around the wellbore stays the same as initial water saturation. For research purposes, it is possible to assume that the non-leak-off damage case satisfies both above conditions. This cumulative production and daily production rate were recorded as a base case. They are shown by blue curves in Figs. 7 and 8.

In contrast to the base case, the leak-off damage case is defined with the following two features: (1) water saturation in the fracture zone increases, and (2) non-wetting phase relative permeability decreases. Production under the leak-off damaged condition will be the actual production obtained after hydraulic fracturing. Formation damage is quantified by comparing productions of the leak-off damage case with the base case. Figures 7 and 8 show production rate and cumulative production comparison between both cases. Cumulative production of leak-off damage cases is 92.8% of the base case or a 7.2% reduction in total production. In the first 3 months of production, the daily gas rate of the leak-off damage case is far less than the base case as the reduction is approximately 21.4% at first and gradually follows up the base case production in the next 2 years. This phenomenon can be explained by the clean-up process. At the beginning of production, water is produced with gas until water saturation in the damaged zone is reduced to *S*_wcrit_ value. After clean-up, the production rates of both cases become nearly equal. This observation indicates that most of the total reduction can be attributed to the early period of production.

**Fig. 9:**
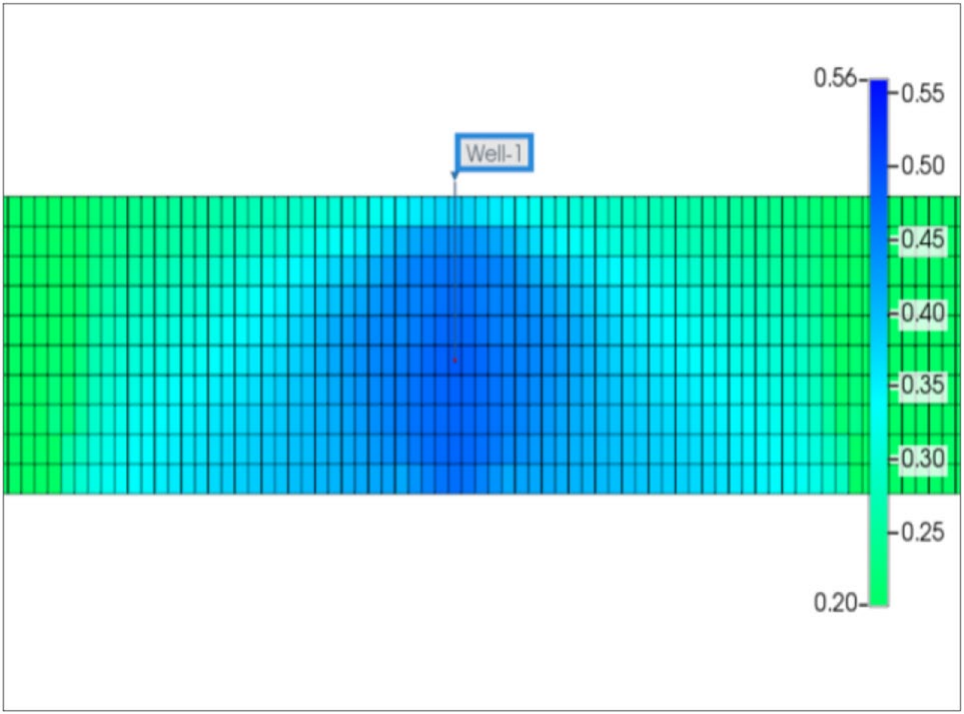
Matrix water saturation distribution in one stage after hydraulic fracturing under normal conditions

**Fig. 10:**
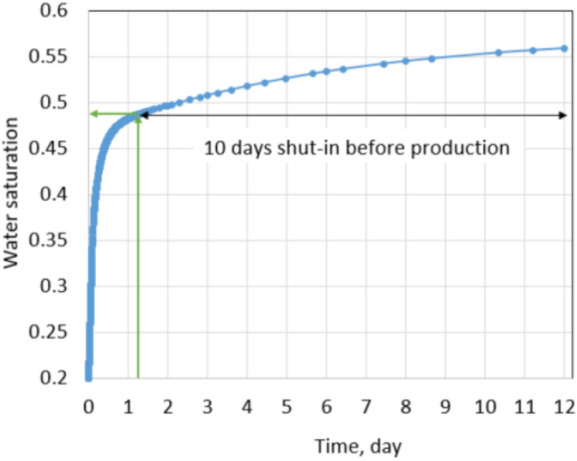
Matrix water saturation around wellbore increase due to fracture fluid invasion under normal conditions

Note that base case production is based on the ideal conditions which do not exist in reality. It represents expected production achieved after the hydraulic fracturing process. The fact is water saturation in the fracture zone always increases under the high injection pressure of the hydraulic fracture process. Figures 9 and 10 show matrix water saturation distribution in one stage after hydraulic fracturing and increment during the shut-in time under leak-off damage. Matrix water saturation in the fracture stage increases from its origin of 0.2 to 0.49 during the hydraulic fracture process. It keeps increasing from 0.49 to 0.56 in 10 days of waiting for production to begin. The increase in the matrix water saturation is caused by slickwater in the fracture system migrating into the matrix. This observation indicates that if the shut-in time is long enough, it will cause additional shut-in damage to the hydraulic fractured well. Generally, matrix water saturation was altered from 0.2 to 0.56, and fracture water saturation increased up to 0.95. It leads to gas relative permeability decreased as shown in Fig. 1 of the literature review section. This relative permeability decrease illustrates why some fractured wells’ performances are much less than expected at the beginning of the production phase.

### Gas Production Impairment Under Extreme Conditions

To determine how severe leak-off damage could be under extreme conditions, the input data as shown in Table 2 were used. In extreme conditions, the *S*_wcrit_ is set at 0.6 instead of 0.4, which means there is a greater risk of water blocking. An extreme water saturation increase will cause a large gas relative permeability decrease; therefore, a severe production impairment. On the other hand, human activities are also the main factors causing formation damage, such as using low conductivity proppant, low drawdown while producing, and long shut-in times. All human activities can lead to more fracture fluid invasion, inefficient fracture permeability, and ineffective clean-up during flowback.

**Fig. 11:**
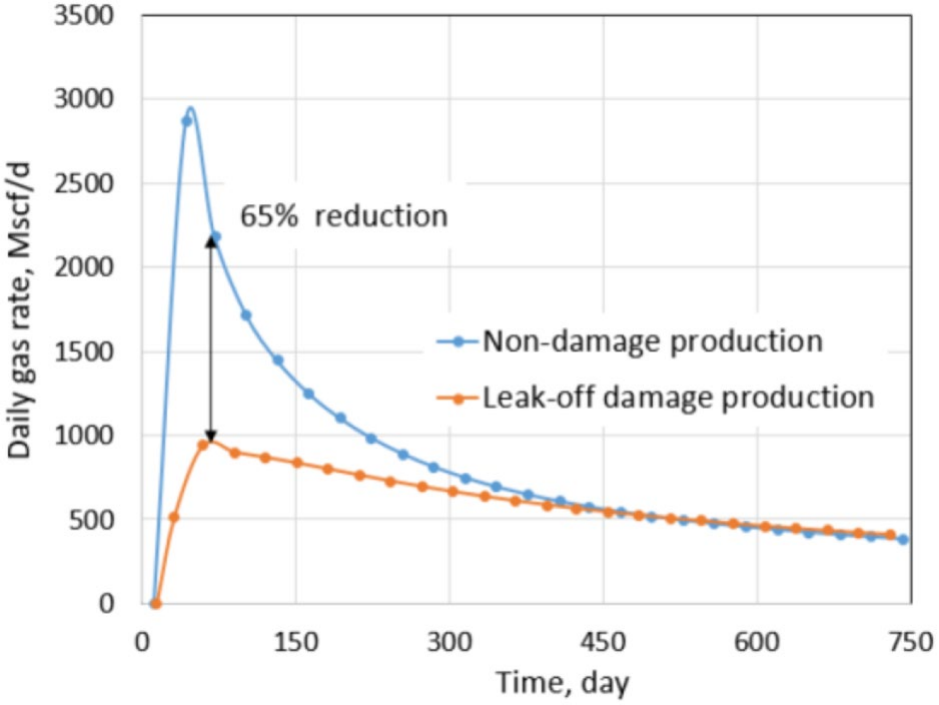
Daily gas rate comparison between non-damaged and leak-off damaged production under extreme conditions

**Fig. 12:**
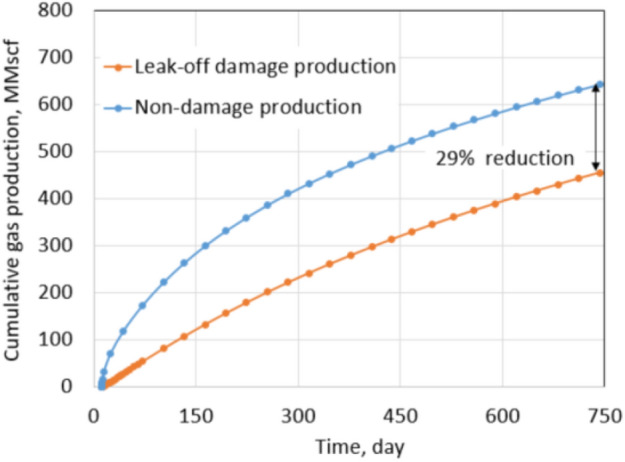
Cumulative production comparison between non-damaged and leak-off damaged under extreme conditions

Similarly, a production comparison between a base case (non-damage case) and a leak-off damage case was conducted. The base case under the extreme conditions has a fracture permeability of 100 mD and no water saturation increase in the fractured zones. It is important to notice that the base case production under extreme conditions and normal conditions are different because of their different residual proppant permeabilities, drawdowns, and *S*_wcrit_ values. However, it is possible to compare formation damage under each condition by looking into the percentage of reduction in the production instead of absolute production. The cumulative production and production rate of the base case under the extreme conditions are shown by the blue curves in Figs. 11 and 12, respectively. Base case production then was compared with production of the leak-off damage case.

Figure 11 shows a daily gas rate comparison between the leak-off damage case and base case. In the first few months, the production rate of the damage case is 65% less than that of the non-damage case. It is a huge reduction in production rate, and it can be explained by an ineffective clean-up. Figure 12 shows a cumulative production comparison between the leak-off damage case and the base case. A significant production impairment of 29% indicates more severe formation damage under the extreme conditions. Once again, most of the total reduction comes from the early production period.

In the normal conditions, the residual proppant permeability is 1000 mD. That permeability is high enough to overcome and mitigate drawbacks of leak-off damage. Moreover, pressure drawdown in the normal conditions is higher than in the extreme conditions, which contributes an important role in the clean-up process. Even though *S*_wcrit_ is an intrinsic property of the reservoir and human efforts cannot change it, operators can improve their production by choosing proppant permeability correctly and increase pressure drawdown by using an artificial lift such as an electric submersible pump (ESP). In the extreme conditions, it takes 450 days for the daily gas rate of the leak-off damage case to be equal to the base case. Meanwhile, that period is only 300 days under the normal conditions. It indicates that the clean-up is more effective in the normal conditions than in the extreme conditions, and it must come from high proppant permeability and high drawdown.

Simulation results showed that the total slickwater injection under normal conditions was 84,000 bbl (13,355 m^3^), but only 20,500 bbl (3260 m^3^) flowed back after 2 years of production. This means the cumulative water flowback was only 24.5% of total water injection, and 75.5% of total water injection was blocked in the reservoir. Meanwhile, the total amount of water injected under extreme conditions was 83,210 bbl (13,230 m^3^), but the cumulative water flowback after 2 years of production was only 8737 bbl (1389 m^3^), which is 10.5% of the total injection. It means a massive amount of water blocking up to 89.5% of total slickwater injection. The root cause is the significant difference between *S*_wi_ and *S*_wcrit_, and it leaves more room for the phase trapping mechanism to occur. On the other hand, using a low conductivity proppant and a poor drawdown of 200 psi makes clean-up inefficient and therefore, reduces recovery.

### Shut-In Damage in Tight Gas Formation

**Fig. 13:**
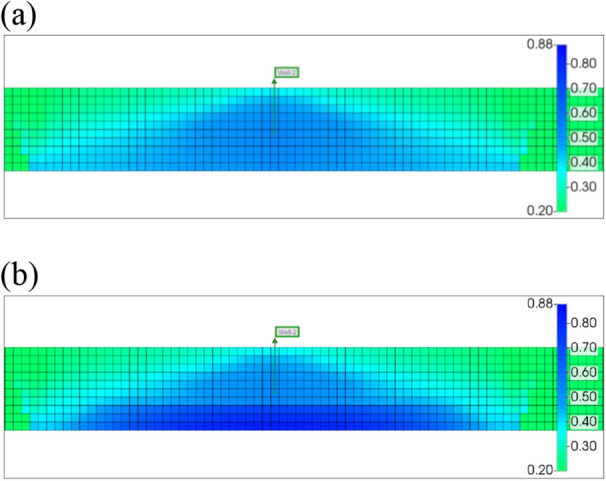
Matrix water saturation distribution in one fracture **a** before shut-in; **b** after shut-in

**Fig. 14:**
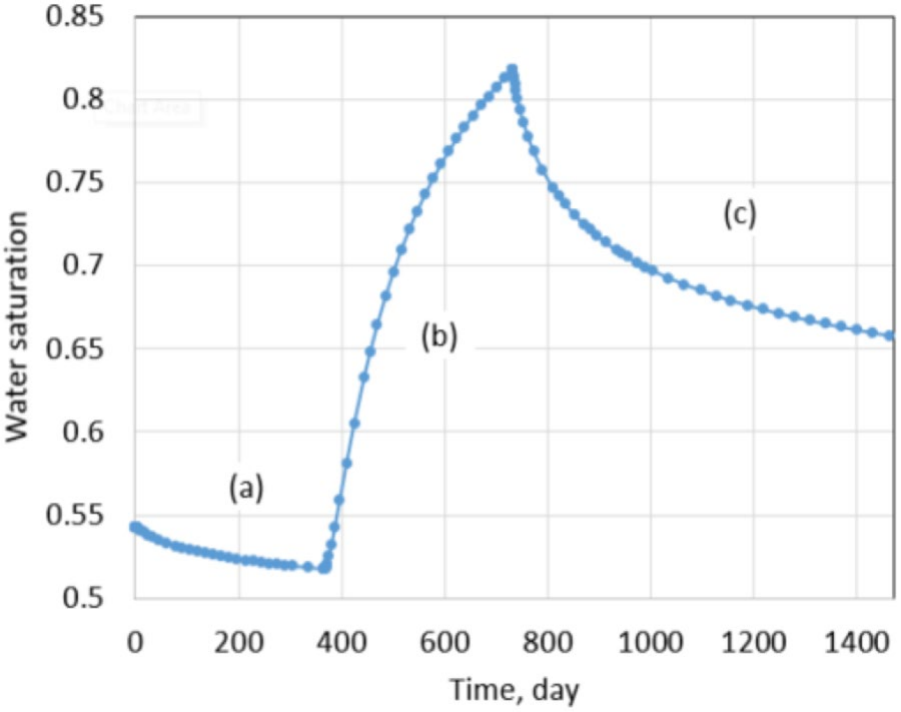
Matrix water saturation at the bottom of the reservoir **a** producing; **b** shut-in; **c** producing

The oil and gas industry has undergone an unprecedented worldwide pandemic. Many wells are shutting down indefinitely. With all this said about leak-off damage, the remaining fracture fluid always exists in the fractures and the matrix. If the producing well is shut down for a long period of time, formation damage will happen as a consequence of water saturation redistribution and proppant embedment. So shut-in damage is an additional formation damage type due to well intervention. To quantify how shut-in affects future recovery, a production comparison method was conducted. The well was produced in 1 year after hydraulic fracturing, then shut in 1 year and kept producing for the next 2 years. Figures 13 and 14 show matrix water saturation redistribution under the normal conditions in 1 year of the shut-in.

During the shut-in time, matrix water saturation at the bottom of the reservoir increases due to the imbibition of the remaining slickwater in the fractures (Wijaya and Sheng 2019, 2020). As shown in Fig. 14, matrix water saturation at the bottom part of the reservoir increases from 0.52 to 0.82 and only decreases when the well is back in production. An increase in the matrix water saturation leads to absolute and relative permeability reductions as presented in Eq. (1) and Fig. 1 in the literature review section.

As fractures are the main flow channels, proppant embedment during shut-in time as discussed in the literature review plays a dominant role in fracture permeability reduction and production decrease due to shut-in damage. Wen et al. (2007) regression for 20/40-mesh suggests that proppant permeability is time-dependent due to embedment, so long-term fracture permeability could become ineffective. However, simulating the percentage of the fracture permeability reduction due to proppant embedment is out of the scope of this study. Based on a study by Bandara et al. (2019) on the percentage of fracture conductivity reduction in shale reservoirs, assumptions of 20% to 75% fracture permeability reductions were made. Production of non-damage formation, which is characterized by no water saturation redistribution and no proppant embedment, was recorded as the base case. Production of shut-in damaged formations was recorded in two scenarios with 20% and 75% fracture permeability reductions, respectively. Formation damage due to shut-in is quantified by comparing the percentage of reduction in production between damage cases and the base case.

Note that the non-damage condition, which is no water saturation redistribution and no proppant embedment, is theoretical and does not exist. During shut-in, remaining slickwater in the fracture system always migrates to the matrix causing a reduction in matrix permeability. In addition, longtime exposure to closure pressure causes long-term fracture permeability reduction in any type of formation. In summary, formation damage due to shut-in is the combination of matrix permeability and fracture permeability reductions.

**Fig. 15:**
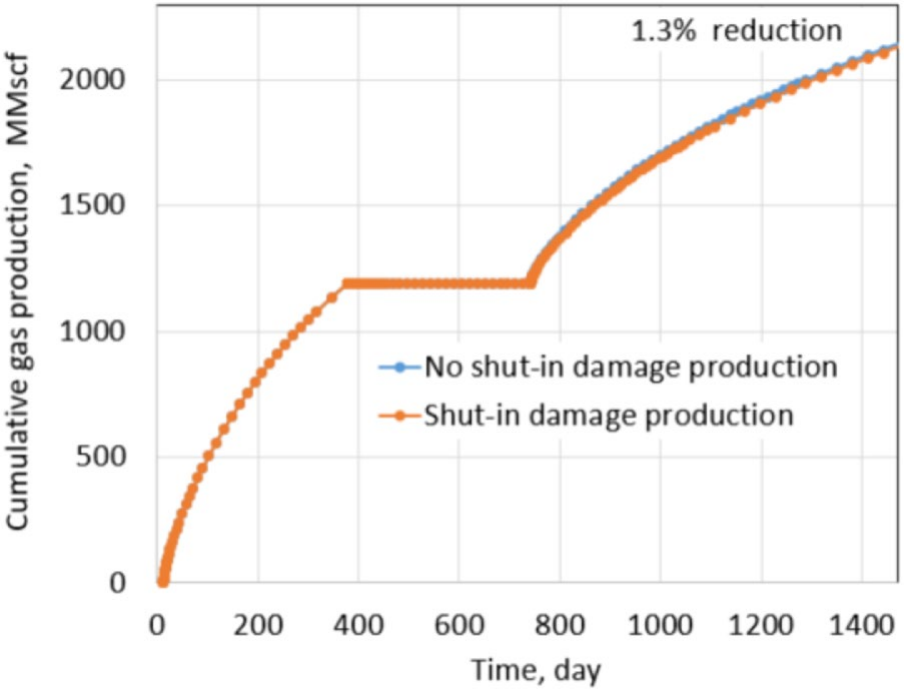
Production comparison with 20% fracture permeability reduction under normal conditions

**Fig. 16:**
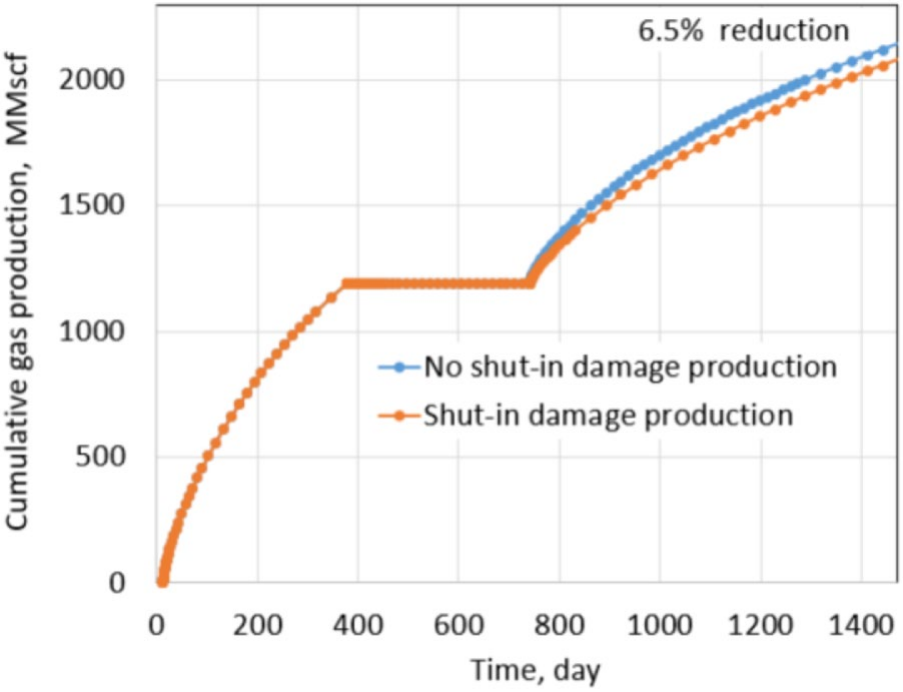
Production comparison with 75% fracture permeability reduction under normal conditions

Figures 15 and 16 show reduction in production due to shut-in damage under normal conditions with fracture permeability reduction of 20% and 75%, respectively.

**Fig. 17:**
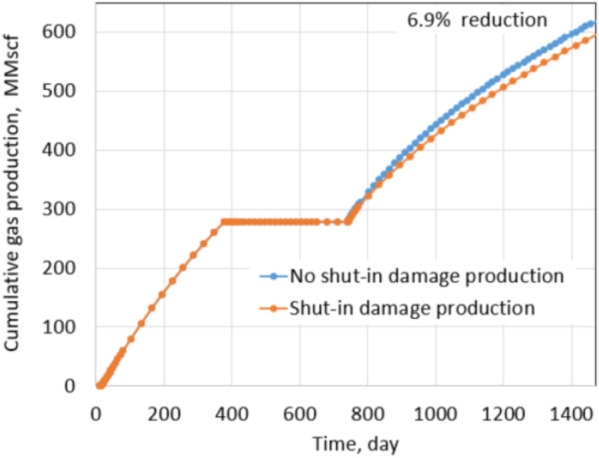
Production comparison with 20% fracture permeability reduction under extreme conditions

**Fig. 18:**
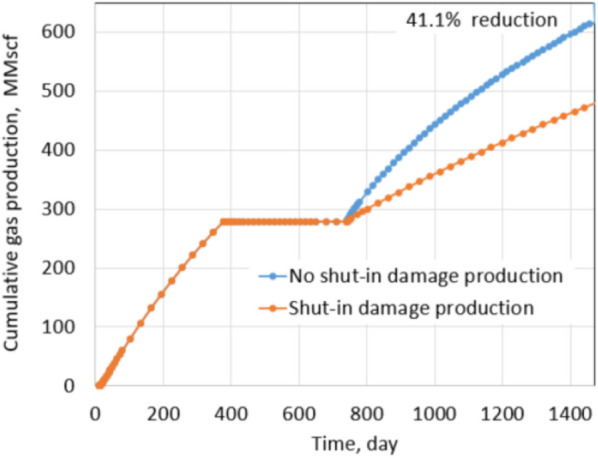
Production comparison with 75% fracture permeability reduction under extreme conditions

Clearly, shut-in damage is not significant under the normal conditions. The reduction in oil production is only 6.5% when fracture permeability reduces up to 75% due to proppant embedment. The exact percentages of fracture permeability reductions are applied for the extreme conditions as shown in Figs. 17 and 18, the percentage of production impairment due to shut-in damage increases significantly to 41.1% when fracture permeability decreases up to 75%. Keep in mind that the fracture permeability reductions used for the normal and extreme conditions are the same, but the production impairment in the extreme conditions is much higher than in the normal conditions. Comparing the normal and extreme conditions clearly indicates the important roles of residual proppant permeability and drawdown pressure in minimizing shut-in damage.

The percentage of fracture permeability reduction due to proppant embedment is assumed and not calculated by the simulation since it depends on many factors such as type of proppant, proppant concentration, and type of formation. However, combining the assumption of fracture permeability reduction and matrix permeability decrease due to water redistribution can explain the mechanism of shut-in damage. Based on simulation results, it proposes two ways to predict production impairment due to shut-in as follows:

- Knowing the type of proppant in use, closure stress, and intended shut-in time, it is feasible to follow the Wen et al. (2007) procedure to obtain a new regression for long-term fracture conductivity reduction. The predicted long-term fracture conductivity reduction is then used in simulation to anticipate production impairment after shut-in.
- Production data of a certain well that experienced a production impairment due to shut-in can be used in a history matching process to estimate the fracture permeability reduction. The result can be used to simulate shut-in damage for other wells in the same reservoir.

### Leak-Off Damage in Shale Oil Formation

#### Oil Production Impairment Under Normal Conditions

To evaluate formation damage due to leak-off, all reservoir and rock properties were kept the same as the normal conditions of tight gas formation and only reservoir fluid was changed from dry gas to black oil in CMG simulation. A horizontal well was drilled at the center of the reservoir and was fractured with 10 stages. To compare between tight gas and shale oil formation, proppant permeability, pump rate, and pumping schedule were kept the same as in the normal conditions. After hydraulic fracturing, the well was produced for 2 years with a constant 600 psi drawdown. (This pressure is also equal to the drawdown of the tight gas simulation under normal conditions.) Similarly, the cumulative oil production and production rate of the no leak-off damage formation (base case) was recorded to compare with leak-off damage case. Once again, base case production is the expected production after fracturing.

**Fig. 19:**
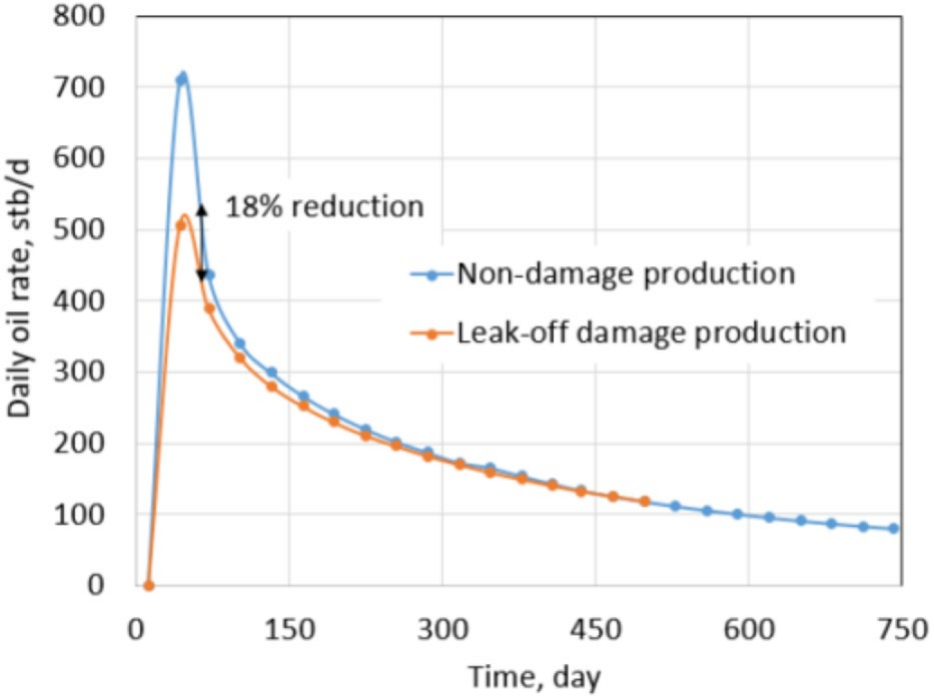
Daily oil rate comparison between non-damaged and leak-off damaged production under normal conditions

**Fig. 20:**
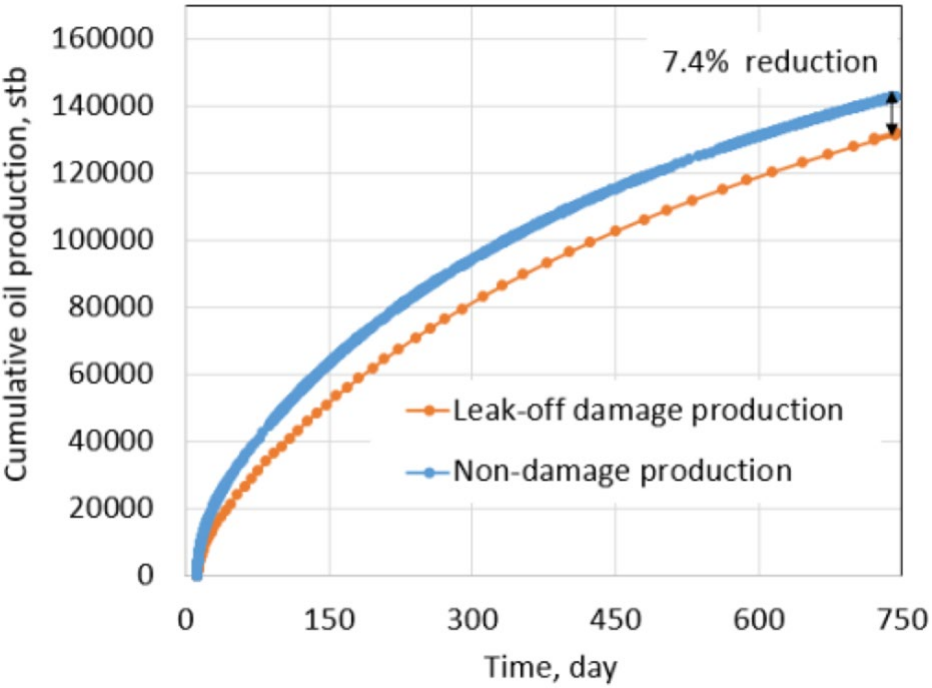
Cumulative oil production between non-damaged and leak-off damaged comparison under extreme conditions

Recall, the leak-off damage case is defined as follows: (1) water saturation in the fracture zone increases and (2) oil permeability decreases as described by the phase trapping mechanism. The production in the leak-off damaged condition will be the actual obtained production. Formation damage of the shale oil simulation is also quantified by comparing the production of the leak-off damage case with the base case. Figures 19 and 20 show reductions in production rate and cumulative production of 18% and 7.4%, respectively. As shown in Fig. 19, the total reduction mostly comes from the first producing period, when the blocking slickwater still exists in the fractures. After a few months of production, the production rate of the damage case is almost equal to the base case because water saturation in the fractures around the wellbore was reduced to *S*_wcrit_.

This result is very similar to the tight gas formation under normal conditions, which has reductions in the production rate and cumulative production of 21.4% and 7.2%, respectively. The slight difference comes from dry gas and black oil fluid properties, which lead to different relative permeability reductions (refer to “Appendix 2”).

### Oil Production Impairment Under Extreme Conditions

In the extreme conditions, *S*_wcrit_ increases from 0.4 to 0.6, residual proppant permeability reduces to 100 mD due to the fracture closure mechanism, and drawdown is 200 psi instead of 600 psi as in the normal conditions. Once again, the base case presents the expected production of an ideal condition where no leak-off occurs. Base case production is then compared with the production of the leak-off damage case. Simulation results showed that the reduced production rate due to leak-off damage at the first 3 months is 38%; meanwhile, it is 18% under the normal conditions. The cumulative reduction is 17.6% compared with 7.4% under the normal conditions. It also takes up to 450 days for the production rate of the leak-off damage case to be equal to the non-damage case, whereas that period under normal conditions is only more than 300 days. It can be explained by: (1) high *S*_wcrit_ leads to a high amount of water blocked and lower oil relative permeability, so the reduction at the beginning of production is high; (2) long clean-up time up to 450 days is the consequence of low residual proppant permeability and poor drawdown of 200 psi.

It is essential to realize that there are two causes for severe formation damage, one from the nature of the reservoir, which is *S*_wcrit_, and one from the human activities which are using low drawdown pressure and low residual proppant permeability. Although the *S*_wcrit_ is out of human control, it is possible to adjust drawdown pressure and residual proppant permeability to mitigate formation damage. If the laboratory test results show a considerable difference between *S*_wi_ and *S*_wcrit_, oil companies should consider a proper proppant permeability and an adequate drawdown pressure accordingly.

### Shut-In Damage in Shale Oil Formation

**Fig. 21:**
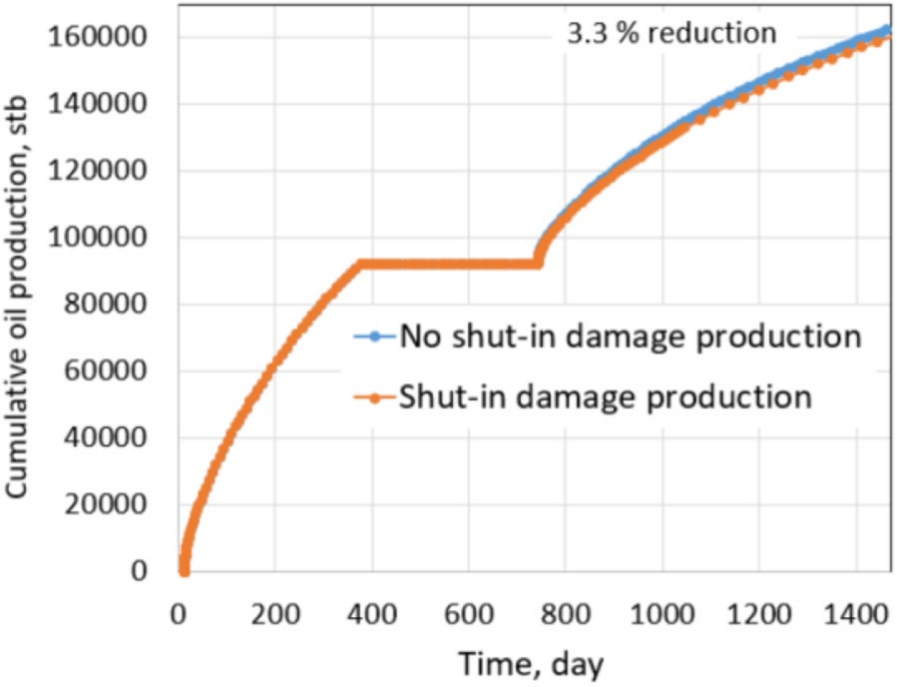
Production comparison with 75% fracture permeability under normal conditions

**Fig. 22:**
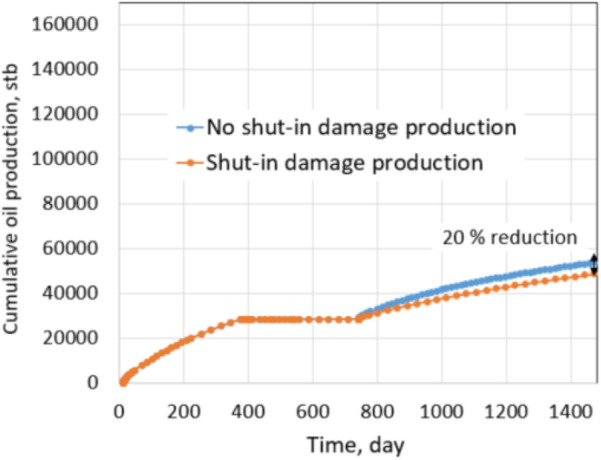
Production comparison with 75% fracture permeability under extreme conditions

As discussed in the tight gas shut-in damage section, potential shut-in damage is caused by: (1) matrix permeability decreases due to water saturation redistribution and (2) fracture permeability reduction due to proppant embedment. The latter term is not measured by this simulation but assumed based on precedent works. The same production comparison as conducted in the tight gas formation was used to evaluate the shut-in impact on future production. The non-damage case is defined by no water saturation redistribution, so there is no matrix permeability decrease, and fracture permeability is not reduced by proppant embedment. It is well noted that the non-damage condition represents expected production after re-opening the well, but it does not exist. The damage case includes both types of permeability reductions in the matrix and fractures. Shut-in damage scenarios under the normal and extreme conditions of shale oil formation were investigated to compare with scenarios of the tight gas formation. Production from damaged cases were simulated with a fracture permeability reduction of 75%. The well was produced in 1 year, then shut in 1 year, and back to production for another 2 years. This shut-in and producing schedule was applied for both non-damage and damage cases, and their productions were compared, as shown in Figs. 21 and 22.

Figure 21 shows the cumulative production comparison of damage and non-damage cases under the normal conditions, and Fig. 22 shows the comparison under the extreme conditions.

Under normal conditions, the impact of shut-in on future production is not noticeable; about 3.3% of cumulative production decreased in the 2 years after shut-in. The shut-in damage under normal conditions of tight gas formation caused 6.9% production impairment in the next 2 years of production, similar to the shale oil shut-in damage under the normal conditions. The difference comes from different fluid properties, relative permeabilities of gas–water and oil–water systems. It leads to matrix water saturation of oil formations altered less than gas formations during shut-in. Thus, the percentage of production impairment in the shale oil formation is less than in the tight gas formation. The same observation is recognized under the extreme conditions. Cumulative reduction in the production of the shale oil is 20% and is 41.1% of the tight gas formation. Clearly, production impairments under the extreme conditions of both types of reservoirs are significant. Note that proppant permeability under the normal conditions and extreme conditions are 1000 mD and 100mD, respectively. Drawdown during production in the normal and extreme conditions is 600 psi and 200 psi, respectively. Thus, comparing the results under the extreme and the normal conditions provides a straightforward acknowledgment of the critical roles of proppant permeability and drawdown pressure.

In a shut-in damage analysis, knowing proppant type, proppant concentration, and its behavior during long bearing-time with closure pressure is the most important key to minimizing formation damage as the consequence of well shut-in. As proposed in the tight gas shut-in damage section, there are two ways to anticipate potential reductions in oil production, which can be applied similarly for the shale oil formation. If the predicted reduction is not tolerant, then the intended shut-in time and type of proppant should be adjusted accordingly. Otherwise, shut-in is not recommended because actual production after shut-in may be far less from expected.

Sensitivities analysis is presented in the next section to determine which key factors that affect each type of formation damage the most and how to improve total recovery.

## Sensitivities Analysis

With all the above discussion, residual proppant permeability and drawdown are the most prominent factors affecting leak-off and shut-in damage. Therefore, this section will concentrate on the analysis of these two factors.

### Effect of Drawdown Pressure

**Fig. 23:**
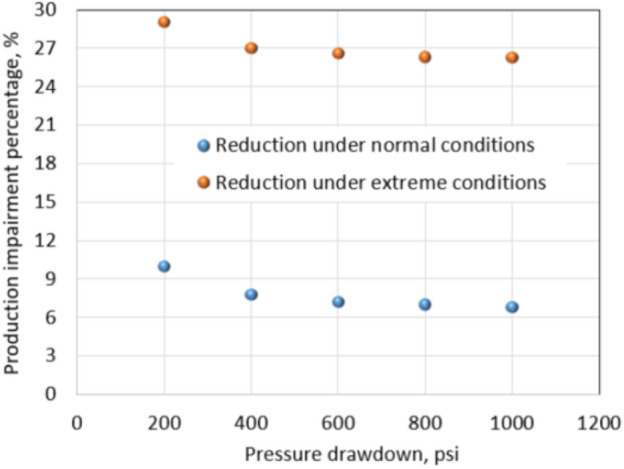
Pressure drawdown versus reductions due to leak-off damage

To evaluate pressure drawdown impact on leak-off damage, the *S*_wcrit_ and residual proppant permeability were retained, and pressure drawdown was varied from 200 to 1000 psi. The tight gas formation under normal conditions was analyzed first and then under extreme conditions was investigated using the same method. With a 1000 psi drawdown, cumulative production in the leak-off damaged formation is 6.8% less than non-damaged formation, meanwhile, that reduction is 10% with a 200 psi drawdown as shown in Fig. 23. In addition, the cumulative production under 1000 psi drawdown is 2.7 Bscf, and it is more than four times that of 200 psi drawdown cumulative production. Clearly, high drawdown pressure helps to mitigate drawbacks of leak-off damage and also increases total cumulative production.

Figure 23 shows percentages of production impairments due to leak-off damage under normal and extreme conditions, corresponding with drawdown variances from 200 to 1000 psi. Under the normal conditions, production impairment percentage reduces from 10% to around 6.8% when the drawdown increases from 200 to 1000 psi. Above 1000 psi, a higher drawdown cannot reduce the percentage of reduction to lower than 6.8%. It is understandable as leak-off fluid always affects oil/gas relative permeability and fracture absolute permeability adversely. In addition, blocking water cannot be removed below *S*_wcrit_.

The drawdown effect under the extreme conditions of the tight gas formation was investigated with the same method as conducted in the normal conditions. Drawdowns vary from 200 to 1000 psi, while *S*_wcrit_ and residual proppant permeability remain unchanged. The percentage of reduction decreases from 29% to 26.3% when drawdown increases from 200 to 1000 psi. Above 1000 psi drawdown, the percentage of production impairment is more or less stable at 26%. This observation shows consistency with the result in the normal conditions. It confirms the effect of drawdown on reducing water saturation in the invaded zone to the value of *S*_wcrit_; afterward, water saturation cannot be reduced below *S*_wcrit_.

The remedial effect of drawdown can be explained by following the phase trapping mechanism. Considering the normal conditions of the tight gas reservoir: Upon hydraulic fracturing, grid blocks around the wellbore were heavily invaded by fracture fluids. The highest value of water saturation is 0.95 as critical gas saturation is 0.05; meanwhile, the highest water saturation in the fracture system shown by CMG was 0.948. This means gas relative permeability in fracture zones around the wellbore was extremely low. When the well is under flowing conditions, water saturation around the wellbore starts to flow back and reduces to the *S*_wcrit_ value of 0.4. In other words, gas relative permeability in the fracture network of grid blocks around the wellbore is improved most likely from 0 to 0.55 as shown in Fig. 1.

This effect is demonstrated once again in Fig. 23 of the extreme conditions. As shown, while pressure drawdown is increasing from 200 to 1000 psi, the percentage of production impairment only improves about 3% (reducing from 29 to 26.3%). It is understandable as the critical water saturation of the extreme conditions is 0.6 instead of 0.4. It indicates that final water saturation under flowing conditions will be 0.6, and gas relative permeability can be improved to a maximum value of only 0.18. In summary, drawdown pressure can help remedy leak-off damage by removing water at the beginning of the production phase. When water saturation in the invaded zone reduces to the *S*_wcrit_, further increasing pressure drawdown will not further reduce the percentage of production impairment. Higher pressure drawdown should give higher absolute recovery; however, the most significant improvement falls into the range of 200 to 1000 psi. To further increase pressure drawdown, artificial lift methods such as ESP should be considered. The initial and operating costs should be considered in comparison with the absolute recovery achieved. This balancing leads to an optimal pressure drawdown which is applicable for each well.

Regarding shut-in damage, drawdown contributes to the removal of blocking slickwater so that the matrix water saturation is not increased during the shut-in time. However, fractures are the main flowing channels, so reduction of fracture permeability due to proppant embedment has a more dominant impact than that of matrix permeability reduction by water blocking. As shown by Wen et al. (2007) and Lacy et al. (1998), fracture conductivity is time-dependent because of proppant embedment. Proppant embedment depends on proppant type, proppant concentration, and exposure time with closure stress. In addition, cyclic loading related to well shut-in and re-opening will accelerate embedment so the long-term fracture conductivity of a shut-in well is much lower. Thus, using a higher drawdown pressure is not effective in mitigating shut-in damage.

Moreover, in some pressure-dependent permeability unconventional reservoirs, very high drawdown reduces production rate as reservoir permeability is sensitive to stress (Nguyen et al. 2020). In general, shut-in damage mostly depends on fracture permeability reduction due to proppant embedment. Therefore, residual proppant permeability and shut-in time is the key factor to reduce shut-in damage.

### Effect of Residual Proppant Permeability

**Fig. 24:**
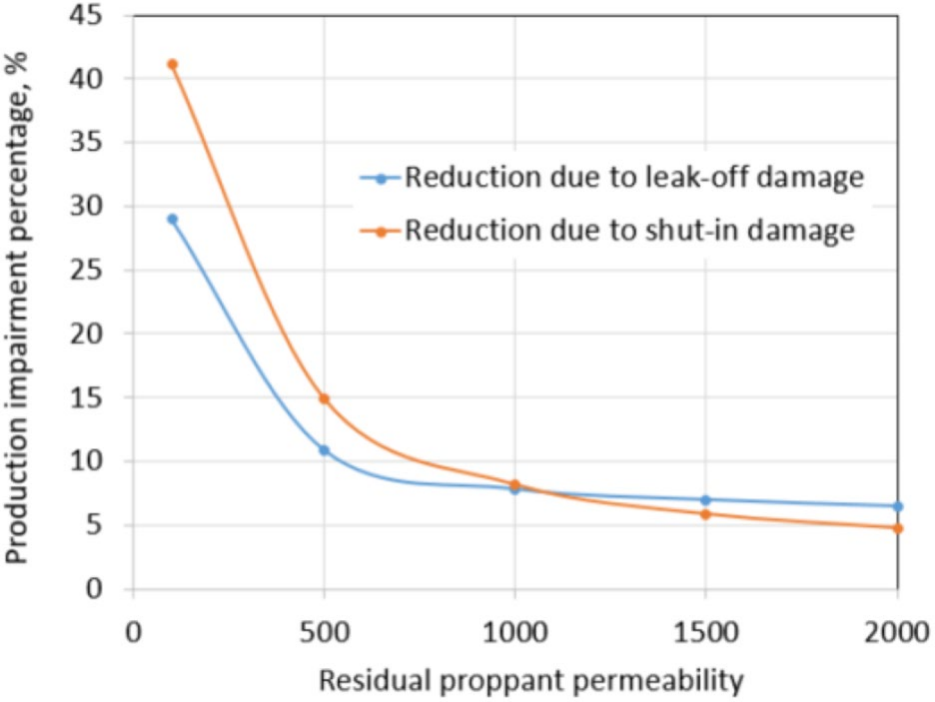
Residual proppant permeability versus reductions under extreme conditions

As discussed previously, leak-off damage is caused by a water-blocking mechanism, and shut-in damage is a consequence of fracture permeability reduction due to proppant embedment. Water blocking can be removed effectively at the beginning of production using high drawdown and high proppant permeability. Regarding leak-off damage, a high residual proppant permeability will help to remove slickwater more effectively during flowback. A high proppant permeability also reduces the risk of water stored in the fracture network migrating into the matrix so that the possibility of shut-in damage is reduced. Fracture permeability reduction during shut-in time can be dealt with by a high residual proppant permeability. With all being said, residual proppant permeability plays the most important role in preventing both types of leak-off and shut-in damage. To evaluate the effect of proppant permeability in improving production, the tight gas formation under extreme conditions of leak-off damage and shut-in damage was investigated by changing residual proppant permeability from 100 to 1500 mD while keeping drawdown and *S*_wcrit_ at 200 psi and 0.6, respectively. Figure 24 shows the effect of residual proppant permeability on the percentage of reduction in production due to leak-off and shut-in damages.

As shown in Fig. 24, production impairment due to leak-off (comparing between damaged and non-damaged production) of tight gas extreme conditions reduces rapidly from 29 to 7% when proppant permeability increases from 100 to 1500 mD. Further increasing of residual proppant permeability to 2000 mD only improves 0.5% of production impairment. Similarly, the percentage of production impairment due to shut-in reduces significantly from 41.1 to 6% when residual proppant permeability increases from 100 to 1500 mD. Increasing proppant permeability from 1500 to 2000 mD only reduces production impairment by 1%. In both types of formation damage, further increasing residual proppant permeability to higher than 2000 mD does not show a significant improvement in the removal of formation damage.

The slopes of curves in Fig. 24 are steeper than in Fig. 23 showing that the residual proppant permeability plays the most important role in improving total hydrocarbon production in both leak-off and shut-in damage scenarios. Sensitivities analysis also indicates an optimal residual proppant permeability in the range of 1500 mD to 2000 mD for this simulation. To be applied for field scale, it is necessary to determine another optimal residual proppant permeability because any variance of reservoir properties will lead to a different optimal value.

## Case Study and Model Validation

The proposed 3D fracture propagation model needs to be validated by using an actual fracture schedule of a multifractured horizontal well and conducting production history matching. In this case study, the validating well was drilled horizontally in 2012 in Eddy County, New Mexico, USA. The productive zone is a tight oil in Delaware formation at true vertical depth (TVD) of 7737 ft (2258 m). Since the thickness of the pay zone is 1800 ft (548 m), this well was fractured with five stages with both vertical and horizontal sections. Core data of porosity and permeability of a nearby well are available, so it was used as the rock properties input for the simulation. Correlation between porosity and permeability of the core data is presented in “Fig. 36 in Appendix 3”. PVT data of the reservoir fluid were measured in 2020 (PDRP 2020) and are shown in Table 3. A black oil model was built based on reported PVT data to implement in the simulation. The initial reservoir pressure and temperature are 2500 psi and 123 F, respectively. Relative permeability curve of the water–oil system is based on the oil shale sample of the Wolfcamp formation (Shiv et al. 2017). “Fig. 37 in Appendix 3” shows the water–oil relative permeability curve with a very high critical water saturation of 0.5 for this shale sample. Reservoir properties of the simulation are shown in Table 3 as follows:

**Table 3:** Reservoir and fluid properties of the case study

Top formation true vertical depth, TVD	1828 ft (2438 m)
Reservoir permeability, *k*	0.01 mD
Reservoir porosity, $\phi^{*}$	0.05
Initial pore pressure, *P*	2500 psi
Reservoir temperature	123 F
Initial water saturation, $S_{{{\text{wi}}}}$	0.2
Critical water saturation, $S_{{{\text{wcrit}}}}$	0.5
Residual oil saturation, $S_{{{\text{or}}}}$	0.07
Young’s modulus, *E*	5E06 psi
Poisson’s ratio, $v$	0.25
Total vertical stress at the well TD, $\sigma_{{\text{V}}}$	8107 psi
Total minimum horizontal stress, $\sigma_{{\text{h}}}$	4369 psi
Bubble point pressure, $P_{b}$	5085 psi
Oil API	42.98
Gas gravity (air = 1)	0.82
Gas–oil ratio	2218 SCF/STB

The well was fractured with five stages, three stages in the vertical and two stages in the lateral section. All stages were fractured using an actual pump rate of 30 bpm. The proppant type is 16/30 White Sand for all stages. Proppant permeability dependent stress is shown in “Fig. 38 in Appendix 3” according to Barree et al. (2003). Pumping time and perforation interval are shown in “Table 6 in Appendix 3”. The well has been producing for 10 years from 2012 to 2022 and is still active.

**Fig. 25:**
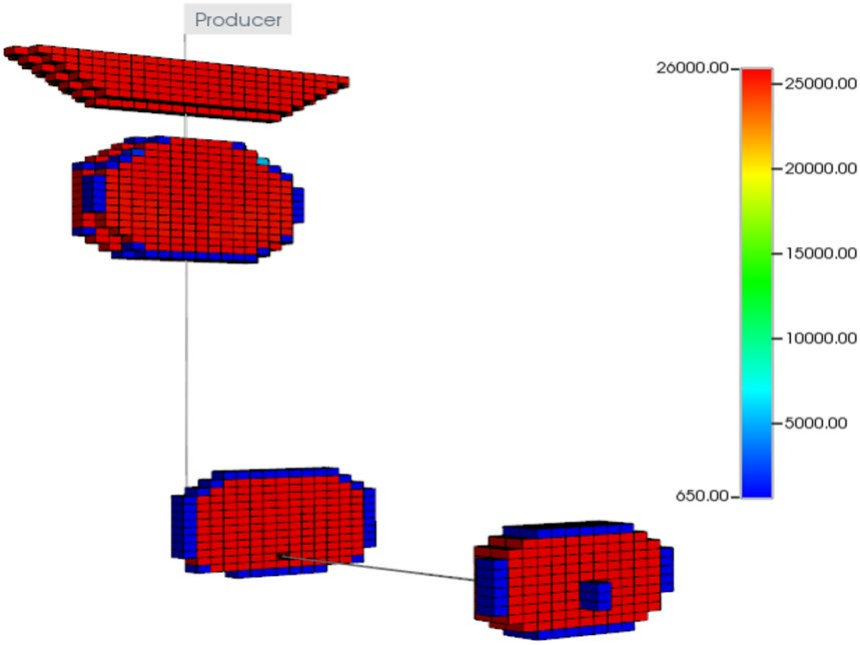
Fractures permeability using 16/30 White Sand

**Fig. 26:**
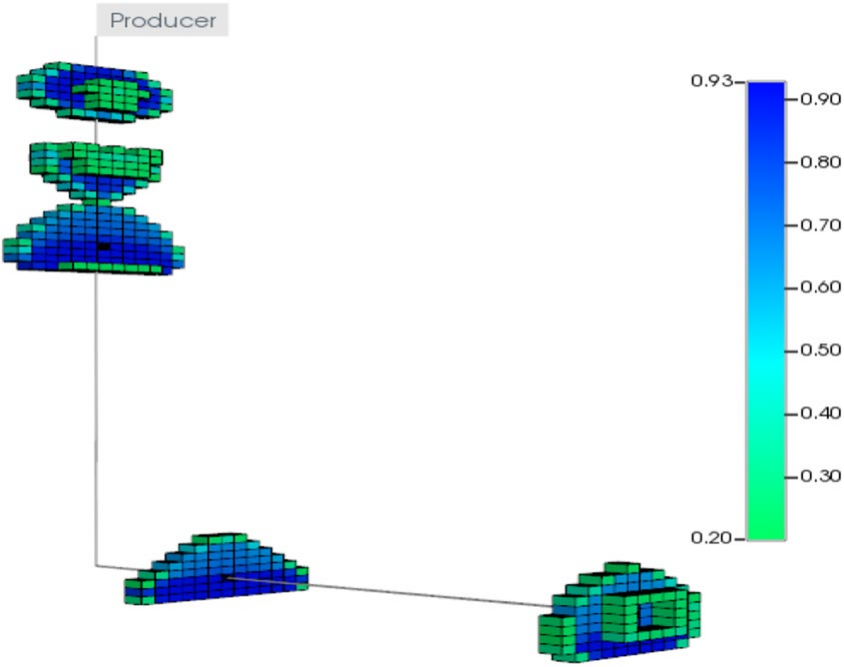
Water saturation alter in fractured zones

The first step of the validation process is to create a reservoir model that can represent the drainage area of the studied well. The reservoir model has uniform block size of 20 ft with 40 grid blocks in the x-direction, 40 blocks in the y-direction, and 90 blocks in the z-direction. The simulated well is located at the depth of 7370 ft TVD and fractured with the reported actual fracture treatment schedule of the studied well. Fracture permeability is set at 26,000 mD for 16/30 White Sand proppant under 8000 psi closure stress (Barree et al. 2003), fracture tip has permeability of 650 mD due to settling and uneven proppant distribution near the tip. Figures 25 and 26 show the fracture permeability and water saturation in the fractured zone, respectively.

History matching was conducted to match the bottom hole pressure (BHP) during hydraulic fracture process first. The purpose of this process is to get the insight into fracture dimensions for the next step of oil production matching. The oil simulation rate then was matched with the observed production data of the studied well. Note that this production is the damaged case, where the reservoir and fracture permeability are decreased due to water saturation increasing in the fracture zone. The damaged fracture and reservoir permeabilities are calculated by Eq. (1). Production history matching was performed by keep changing uncertain parameters, including fracture dimensions, proppant permeability, near fracture tip permeability, reservoir permeability and bottom hole flowing pressure, until an acceptable match is achieved. Table 4 shows the fitting parameters to get the best fit in this case study.

**Table 4:** Fitting parameters

Flowing bottom hole pressure	1750 psi
Proppant permeability	26,000 mD
Near fracture tip permeability	650 mD
Reservoir permeability	0.129 mD

**Fig. 27:**
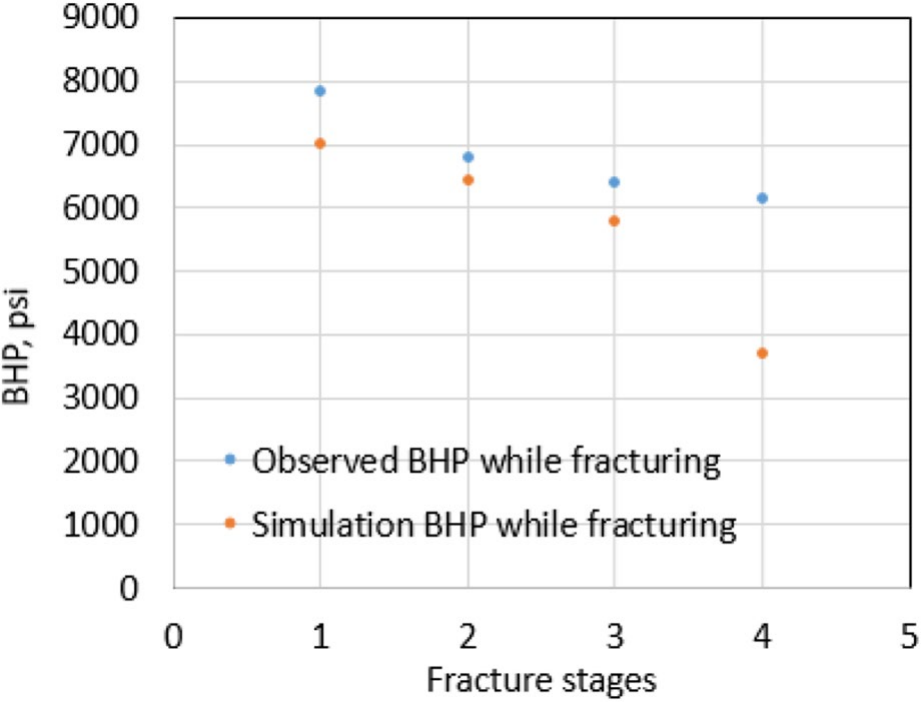
Matching of BHP while hydraulic fracturing

**Fig. 28:**
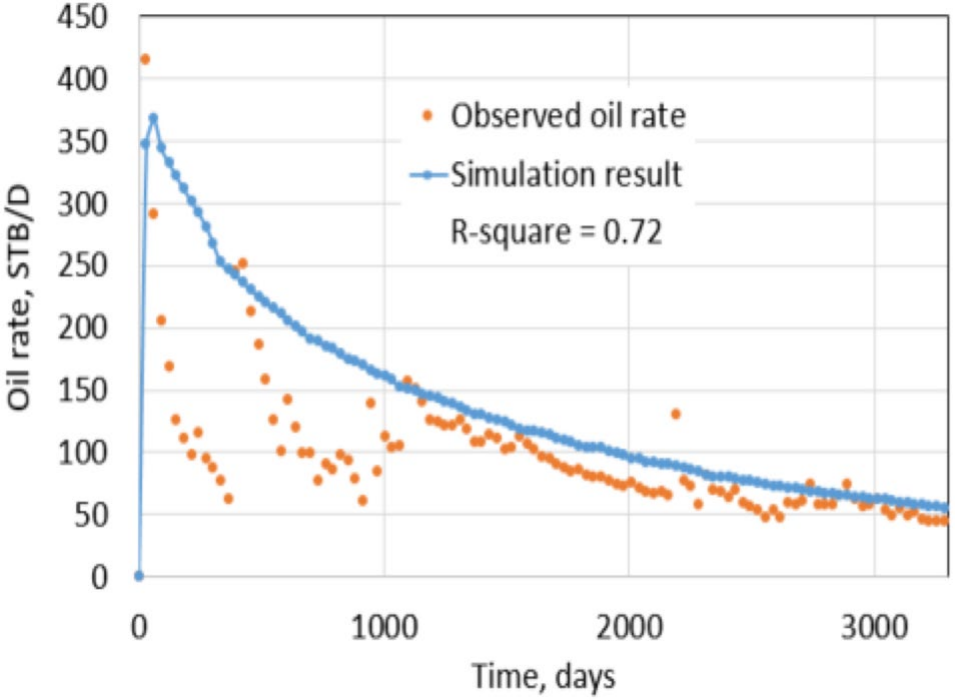
Oil production matching result

Figures 27 and 28 show the bottom hole pressure matching during hydraulic fracturing and the daily oil rate matching of production in 10 years. The covariance (*R*^2^ value) is 0.72, indicating a good match between historical oil data and simulation result. After matching, the model can be used to predict leak-off damage by comparing damaged with non-damaged production.

**Fig. 29:**
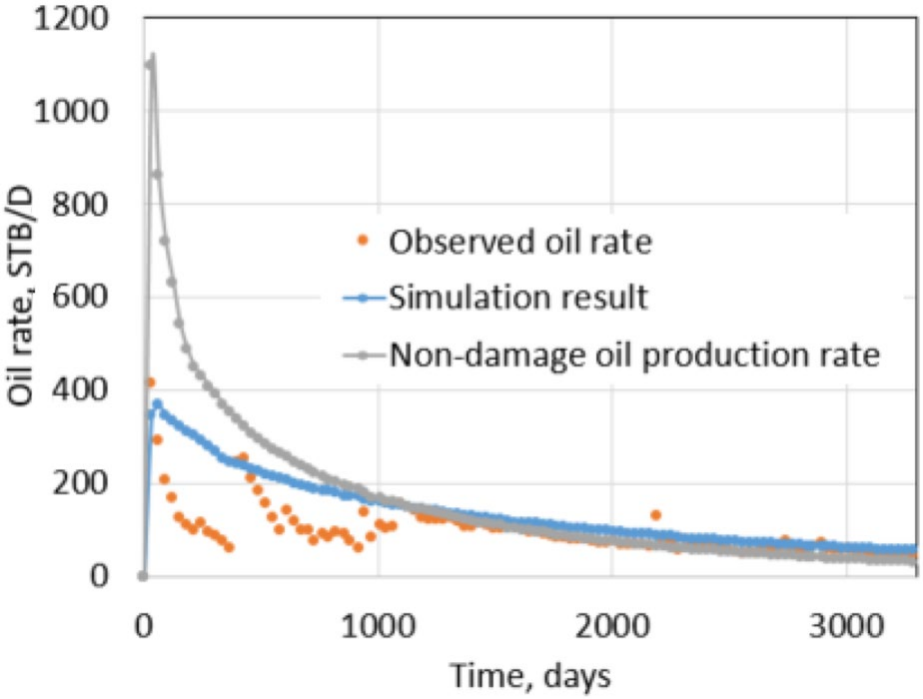
Daily oil rate comparison

**Fig. 30:**
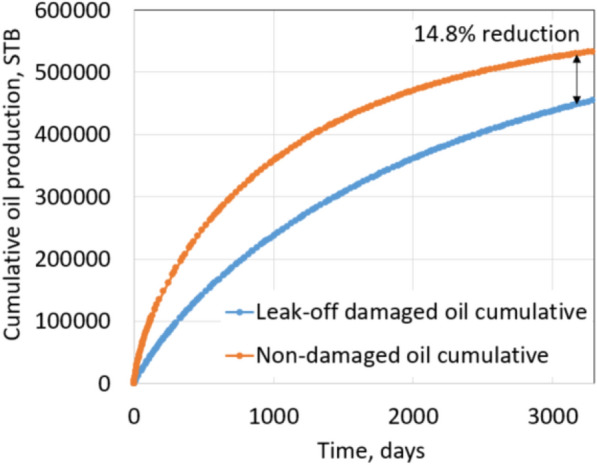
Cumulative production comparison

**Fig. 36:**
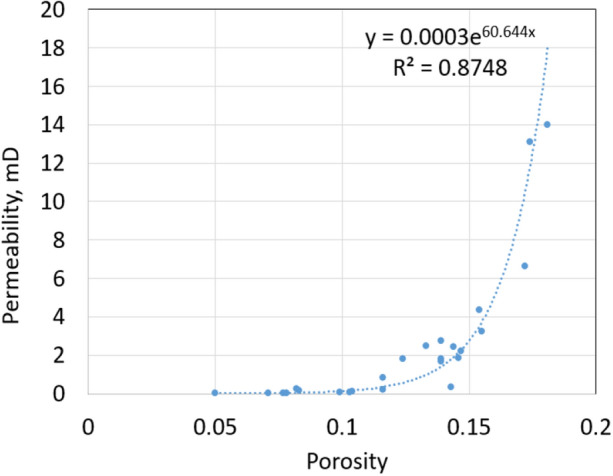
Porosity and permeability core data

**Fig. 37:**
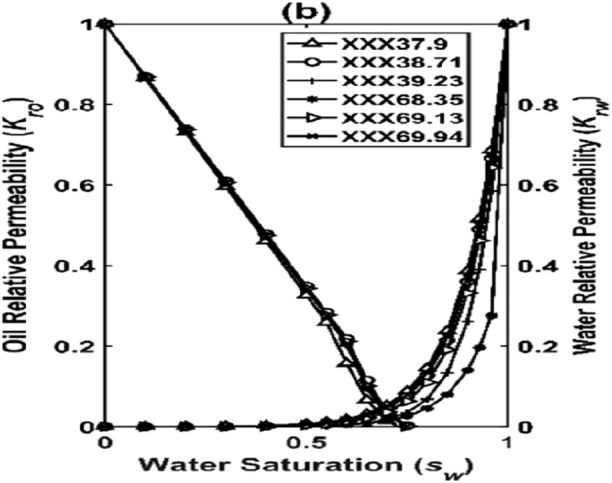
Relative permeability curve (Shiv et al. 2017)

**Fig. 38:**
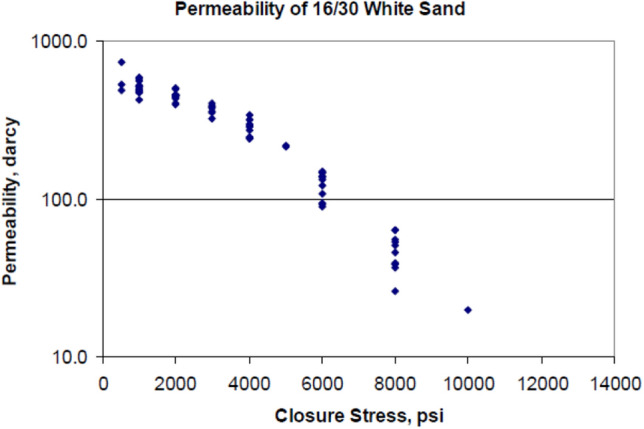
Permeability versus stress for 16/30 White Sand (Barree et al. 2003)

Non-damaged production is achieved by using the non-damage fracture and reservoir permeabilities for the matched model. Figures 29 and 30 show the comparison between damaged and non-damaged rates and cumulative productions. In this case study, the cumulative production after 2 years of the non-damaged case is 533,000 STB, while the leak-off damaged case is 454,000 STB. It is a reduction of 14.8% due to leak-off damage.

## Limitations of the Model

The initial limitation of the model comes from the assumption of homogeneous and isotropic rock properties. Due to the lack of field data, rock properties used in the simulation, such as permeability, porosity, Poisson’s ratio, and Young’s modulus, are hypothetical and typical for tight formations. Since the simulated reservoir is isotropic, the model results in uniform and symmetric fracture propagation. However, the fracture shape in reality is asymmetrical due to reservoir heterogeneity. Second, the model does not consider the proppant transportation. It does not calculate the fracture width but assigns the fracture permeability based on the effective stress threshold. Once the effective stress of the formation rock reduces below tensile strength, the fracture is created. Since no proppant transportation is simulated, the fracture permeability is uniform and equally distributed in the stimulated zone. In fact, fracture permeability will alter with proppant concentration, settling, damage, etc. The model results can be improved if several well logs available to create a structural map of the interested location. Porosity and permeability of the heterogeneous formation then are populated using continuous porosity log and permeability core data to achieve a more realistic fracture propagation in the reservoir.

## Conclusions

Fracture fluid invasion always exists with multi-fractured horizontal wells in tight reservoirs because of differential pressure during treatments and waiting time on fractures closure. After hydraulic fracturing, a significant amount of fracture fluid blocked in the fracture zone is not removable due to the high *S*_wcrit_ and phase trapping mechanism. This blocking fluid reduces hydrocarbon relative permeability and reservoir permeability. In addition, blocking fluid in the fracture network migrates into the matrix and causes matrix permeability reduction during well shut-in. Matrix permeability and fracture permeability reductions, caused by proppant embedment, are the root causes of formation damage due to well shut-in.

Although formation damage due to fracture fluid invasion always exists in a multi-fractured horizontal well, production from damaged formations can be improved with comprehension of reservoir properties, especially *S*_wcrit_ and fluid properties. Shut-in damage can be dealt with by using a proper proppant type. Several methods to improve the production of damage formations are as follows:

- Determine optimal residual proppant permeability before choosing a proppant pack for hydraulic fracturing. Optimal residual proppant permeability should be considered in balancing between the percentage of production improvement and the initial cost of the proppant pack. Choosing optimal proppant permeability is a pre-treatment method and is highly effective in preventing both leak-off and shut-in damages.
- Post-treatment methods include optimal pressure drawdown and reducing shut-in time. Pressure drawdown shows its high efficiency in increasing production due to leak-off damage; however, this method is not highly effective in removing shut-in damage. The reason is that shut-in damage is dominated by fracture permeability reduction due to proppant embedment.
- Once shut-in damage happens, it means proppant permeability has been damaged. Increasing drawdown does not improve productivity, it even reduces production in some unconventional pressure-dependent-permeability reservoirs. Thus, shut-in damage should be prevented in advance by choosing high proppant conductivity and reducing shut-in time. Instead of shutting-in a specific well for a long time, shut-in time should be divided into several wells in the field to minimize the risk of proppant embedment due to longtime exposure with closure pressure. A high proppant permeability can also help to sustain long-term fracture permeability when the well is shut and hence minimize the risk of production reduction after shut-in.

Two methods to obtain fracture permeability reduction after well shut-in are proposed, including: (1) generating a correlation between long-term fracture conductivity and type of proppant in use; (2) using history matching technique to find fracture permeability reduction after shut-in time. As stated, simulation of fracture reduction was not conducted in this paper. It is open for a potential future work, which can focus on simulation of the fracture permeability behavior and reduction in considering proppant transportation, concentration, and embedment due to long stress-bearing time. Production of shut-in wells then can be predicted based on simulated fracture permeability reduction.

## Figures and Tables

**Fig. 31:**
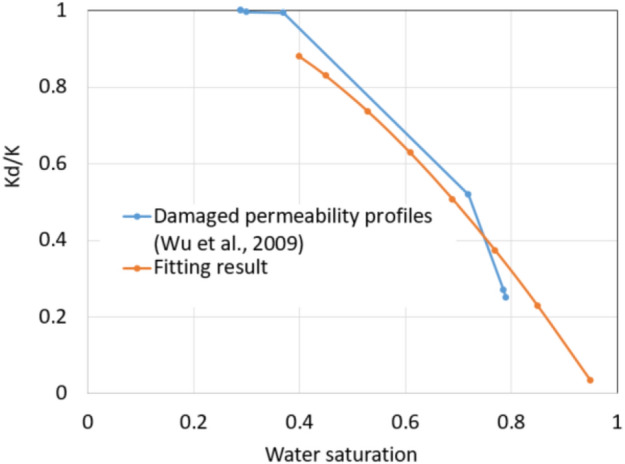
Fitting damaged permeability profile to determine $\alpha$ and $b_{{\text{w}}}$ coefficients

**Fig. 32:**
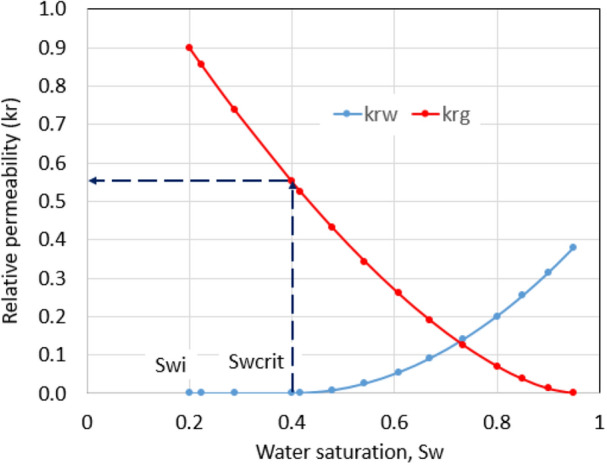
Gas relative permeability used in tight gas base case simulation

**Fig. 33:**
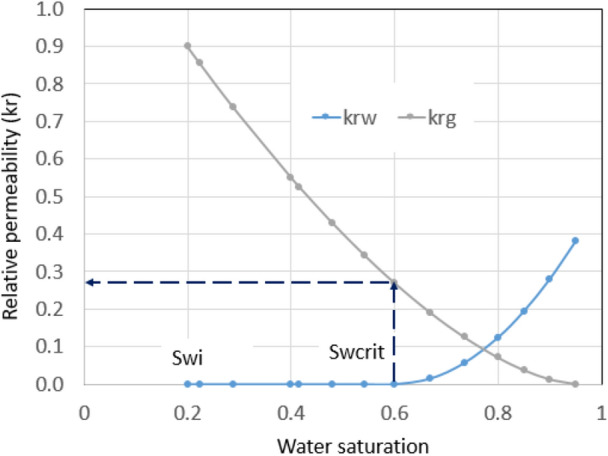
Gas relative permeability used in tight gas extreme case simulation

**Fig. 34:**
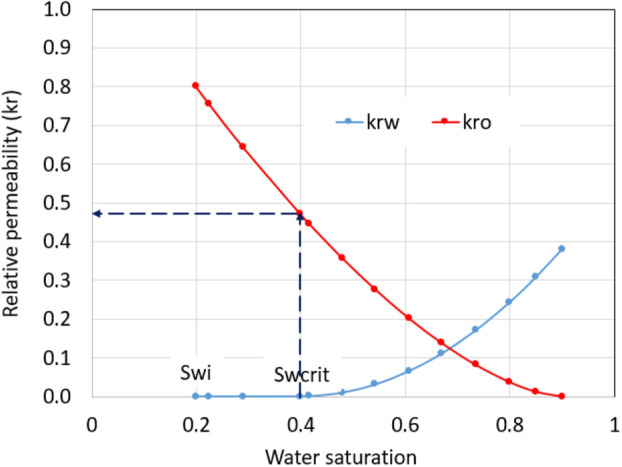
Oil relative permeability used in shale oil base case simulation

**Fig. 35:**
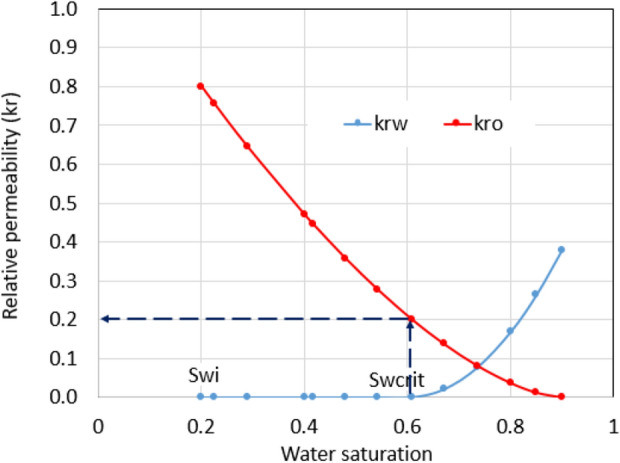
Oil relative permeability used in shale oil extreme case simulation

## Appendix 1

See Fig. 31, Table

**Table 5:** Fitting result using Eq. (1) to determine $\alpha$ and $b_{{\text{w}}}$ coefficients

Water saturation and damaged permeability profile (Wu et al. 2009)				Fitting result using Eq. (1) to determine $\alpha$ and $b_{{\text{w}}}$ coefficients	
*S*_w_	*k*_d_	*k*	*k*_d_/*k*	*S*_w_	*k*_d_/*k*
0.29	0.52	0.52	1	0.4	0.88
0.29	0.52	0.52	1	0.45	0.829
0.3	0.518	0.52	0.996	0.53	0.735
0.37	0.27	0.52	0.994	0.61	0.627
0.72	0.27	0.52	0.519	0.69	0.506
0.785	0.14	0.52	0.269	0.77	0.373
0.79	0.13	0.52	0.25	0.85	0.228
				0.95	0.033

^5^.

## Appendix 2

See Figs. 32, 33, 34 and 35.

## Appendix 3

See Figs. 36, 37 and 38, Table

**Table 6:** Actual fracture treatment of the studying well

	Stage 1	Stage 2	Stage 3	Stage 4	Stage 5
Pumping time, min	100	100	135	65	90
Pump rate, bpm	30	30	20	25	25
Perforation interval, ft	7940–7950	7530–7540	6480–6500	6230–6240	6075–6085
BHP while pumping, psi	7840	6196	6406	6160	N/A

6.

## List of symbols

**B**: Body force/ unit mass of solid grain, m/s^2^
$b_{w}$: Exponent of relative permeability
*C*: Constant of long-term fracture conductivity equation, µm^2^/cm^−^^1^
**C**: Tangential stiffness tensor, psi
*E*: Young’s modulus, MPa
$f_{{{\text{rs}}}}$: Tensile strength, psi
$f_{{\text{s}}}$: Probability density function for pore size-distribution under effective stress
$F_{{{\text{RCD}}}}$: Long-term fracture conductivity, µm^2^/cm^−^^1^
**g**: Gravitational acceleration, m/s^2^
**I**: Unit tensor
*k*: Absolute permeability, mD
**k**: Absolute permeability tensor, m^2^
$k_{{{\text{ccf}}}}$: Closure permeability, mD
$k_{{\text{d}}}$: Damage permeability in the invaded zone, mD
$k_{{{\text{hf}}}}$: Proppant permeability, mD
$k_{{{\text{rcf}}}}$: Residual fracture permeability, mD
$k_{{{\text{rg}}}}$: Gas relative permeability
$k_{{{\text{ro}}}}$: Oil relative permeability
$k_{{{\text{rw}}}}$: Water relative permeability
$L_{{\text{s}}}$: Tortuous flow path under effective stress, m
*N*: Number of pores in a unit cell
*p*: Fluid pressure, Pa
*P*: Initial pore pressure, psi
$P_{{\text{b}}}$: Bubble point pressure, psi
$Q_{{\text{f}}}$: Mass flow rate of fluid per unit volume
*r*: Inner radius of a certain pore, m
$r_{{{\text{cs}}}}$: Critical pore radius under effective stress, m
$r_{{{\text{maxs}}}}$: Maximum pore radius under effective stress, m
$r_{{{\text{mins}}}}$: Minimum pore radius under effective stress, m
$r_{{\text{s}}}$: Inner radius of a certain pore under effective stress, m
$S_{{{\text{gc}}}}$: Critical gas saturation
$S_{{{\text{or}}}}$: Residual oil saturation
$$S_{{\text{w}}}$$: Water saturation
$S_{{{\text{wcrit}}}}$: Critical water saturation
$S_{{{\text{wi}}}}$: Initial water saturation
*t*: Time, day
$T_{0}$: Tensile rock strength, psi
**u**: Displacement vector, m/s
**v**: Velocity vector, m/s
$V_{{\text{b}}}$: Current bulk volume, m^3^
$V_{{\text{b}}}^{0}$: Initial bulk volume, m^3^
$V_{{\text{p}}}$: Current pore volume, m^3^
$V_{{{\text{total}}}}$: Volume of the total pore space, m^3^
$V_{{{\text{wcrit}}}}$: Volume of the immobile water, m^3^
$\alpha$: Formation damage factor
**α**: Biot’s constant
$\beta$: Power-law index
$${{\varvec{\upvarepsilon}}}$$: Strain tensor
$$\varepsilon_{{\text{V}}}$$: Volumetric strain
$\eta$: Stationary water coefficient
*µ*: Fluid viscosity, Pa.s
$$v$$: Poisson’s ratio
$\rho_{{\text{f}}}$: Fluid density, kg/m^3^
$\rho_{r}$: Solid grain density, kg/m^3^
${{\varvec{\upsigma}}}$: Total stress tensor, psi
${\mathbf{\sigma^{\prime}}}$: Effective stress tensor, psi
$\sigma_{1}^{^{\prime}}$: Maximum effective stress, psi
$\sigma_{3}^{^{\prime}}$: Minimum effective stress, psi
$\sigma_{{\text{h}}}$: Total minimum horizontal stress, psi
$\sigma_{{\text{h}}}^{^{\prime}}$: Effective minimum horizontal stress, psi
$\sigma_{{\text{n}}}^{^{\prime}}$: Effective normal stress, psi
$\sigma_{{{\text{ob}}}}^{^{\prime}}$: Effective overburden stress, MPa
$\sigma_{{\text{V}}}$: Total vertical stress, psi
$\sigma_{{\text{v}}}^{^{\prime}}$: Effective vertical stress, psi
$$\phi$$: True porosity
$$\phi^{*}$$: Reservoir porosity

## References

[CR1] Ahamed MAA, Perera MSA (2021). Pressure-dependent flow characteristics of proppant pack systems during well shut-in and the impact of fine invasion. J Natl Gas Sci Eng.

[CR2] Bandara KMAS, Ranjith PG, Rathnaweera TD (2019). Improved understanding of proppant embedment behavior under reservoir conditions: a review study. Powder Technol.

[CR3] Barree RD, Cox SA, Barree VL, Conway MW (2003). Realistic assessment of proppant pack conductivity for material selection. SPE.

[CR4] Chen Y, Zhang C, Zhu L (2017). A fractal irreducible water saturation model for capillary tubes and its application in tight gas reservoir. J Petrol Sci Eng.

[CR5] Chen M, Li P, Kang Y, Gao X, Yang D, Yan M (2021) Application of heat treatment to prevent fracturing fluid-induced formation damage and enhance matrix permeability in shale gas reservoirs. In: SPE-205591-MS, prepared for presentation at the SPE/IATMI Asia Pacific Oil & Gas Conference and Exhibition held virtually on 12–14 October, 2021. 10.2118/205591-MS

[CR6] Cheng B, Li J, Li J, Su H, Tang L, Yu F, Jiang H (2022). Pore-scale formation damage caused by fracturing fluids in low-permeability sandy conglomerate reservoirs. J Petrol Sci Eng.

[CR7] Cuss R, Waters CN, Hennissen J, Wiseall AC (2015) Hydraulic fracturing: a review of theory and field experience. British Geological Survey open report OR/15/066

[CR8] Ding DY, Langouet H, Jeannin L (2013) Simulation of fracturing-induced formation damage and gas production from fractured wells in tight gas reservoirs. SPE-153255-PA. 10.2118/153255-PA

[CR9] Garduno J, King G (2020) Managing risk and reducing damage from well shut-ins. J Pet Technol

[CR10] Gou X, Guo J, Lu C, Chen S (2017). A new method of proppant embedment experimental research during shale hydraulic fracturing. Electron J Geotech Eng.

[CR11] He W, Liu Z (2021). Numerical simulation of formation damage by drilling fluid in low permeability sandstone reservoirs. J Pet Explor Prod.

[CR12] Huang J, Safari R, Perez O, Fragachan FE (2019) Reservoir depletion-induced proppant embedment and dynamic fracture closure. In: SPE-195135-MS, prepared for presentation at the SPE Middle East Oil and Gas Show and Conference held in Manama, Bahrain. 10.2118/195135-MS

[CR13] Labuz JF, Zang A (2012). Mohr-Coulomb failure criterion. Rock Mech Rock Eng.

[CR14] Lacy LL, Rickards AR, Bilden DM (1998) Fracture width and embedment testing in soft reservoir sanstone. SPE-36421-PA. 10.2118/36421-PA

[CR15] Li H, Liu Z, Jia N, Chen Xu, Yang J, Cao L, Li B (2021). A new experimental approach for hydraulic fracturing fluid damage of ultradeep tight gas formation. Geofluids.

[CR16] Liang T, Gu F, Yao E, Zhang L, Yang K, Liu G, Zhou F (2017). Formation damage due to drilling and fracturing fluids and its solution for Tight Naturally Fractured Sandstone Reservoirs. Geofluids.

[CR17] Miskimins JL, Holditch SA, Veatch RWJr (2019) Hydraulic fracturing: fundamentals and advancements. SPE Monograph Series Vol 12

[CR18] Nguyen TC, Pande S, Bui D, Al-Safran E, Nguyen HV (2020). Pressure dependent permeability: unconventional approach on well performance. J Petrol Sci Eng.

[CR19] Qutob H, Byrne M (2015) Formation damage in tight gas reservoirs. In: SPE-174237-MS, prepared for presentation at the SPE European Formation Damage Conference and Exhibition held in Budapest, Hungary. 10.2118/174237-MS

[CR20] PDRP (2020) New Mexico Institute of Mining and Technology, 2020: PVT analysis of surface sample from Sandy Federal #3 Well

[CR21] Shiv PO, Siddharth M, Ali T, Carl S, Chandra R (2017). Relative permeability estimates for Wolfcamp and Eagle Ford shale samples from oil, gas and condensate windows using adsorption-desorption measurements. Fuel.

[CR22] Su YL, Fu JG, Li L, Wang WD, Zafar A, Zhang M, Ouyang WP (2020). A new model for predicting irreducible water saturation in tight gas reservoirs. Pet Sci.

[CR23] Tran D, Settari A, Nghiem L (2002) New iterative coupling between a reservoir simulator and a geomechanics module. In: SPE/ISRM 78192, prepared for presentation at the SPE/ISRM Rock Mechanics Conference held in Irving, Texas, USA. 10.2118/88989-PA

[CR24] Tran D, Buchanan L, Nghiem L (2008) Improved gridding technique for coupling geomechanics to reservoir flow. In: SPE-115514, prepared for presentation at the 2008 SPE annual technical conference and exhibition held in Denver, Colorado, USA. 10.2118/115514-PA

[CR25] Tran D, Nghiem L, Buchanan L, (2009a) Aspects of coupling between petroleum reservoir flow and geomechanics. In: ARMA 09-089, presented at Asheville 2009a, the 43rd U.S. Rock Mechanics Symposium and 4th U.S.—Canada Rock Mechanics Symposium, held in Asheville, North Carolina, USA

[CR26] Tran D, Shrivastava V, Nghiem LX, Kohse BF (2009b) Geomechanical risk mitigation for CO_2_ sequestration in saline aquifers. In: SPE-125167, prepared for presentation at the 2009b SPE Annual Technical Conference and Exhibition held in New Orleans, Louisiana, USA. 10.2118/125167-MS

[CR27] U.S. Energy Information Administration, Annual Energy Outlook 2021. www.eia.gov/outlooks/archive/aeo21

[CR28] Wen Q, Zhang S, Wang L (2007). The effect of proppant embedment upon the long-term conductivity of fractures. J Petrol Sci Eng.

[CR29] Wijaya N, Sheng JJ (2020) Shut-in effect in removing water blockage in shale-oil reservoirs with stress-dependent permeability considered. SPE-195696-PA. 10.2118/195696-PA

[CR30] Wijaya N, Sheng JJ (2019). Comparative study of well soaking timing (pre vs. post flowback) for water blockage removal from matrix-fracture interface. Petroleum.

[CR31] Wu Z, Vaidya RN, Suryanarayana PV (2009) Simulation of dynamic filtrate loss during the drilling of a horizontal well with high-permeability contrasts and its impact on well performance. SPE-110677-PA. 10.2118/110677-PA

